# A novel fragmented anode biofilm microbial fuel cell (FAB–MFC) integrated system for domestic wastewater treatment and bioelectricity generation

**DOI:** 10.1186/s40643-021-00442-x

**Published:** 2021-11-13

**Authors:** Tesfalem Atnafu, Seyoum Leta

**Affiliations:** 1 Center for Environmental Science, Addis Ababa University, Addis Ababa, Ethiopia; 2 Department of Biological Science, College of Natural Sciences, Mettu University, Mettu, Ethiopia

**Keywords:** Microbial fuel cell, Fragmented anode biofilm reactor (FAB), Electroactive biofilm (EAB), Anode surface area, Microbial electrode jacket dish (MEJ-dish)

## Abstract

**Background:**

The critical MFC design challenge is to increase anode surface area. A novel FAB–MFC integrated system was developed and evaluated for domestic wastewater treatment. It was operated in fed-batch flow mode at 1–3 days of HRT with 755 mg/L COD_IN_ and 0.76 kg-COD/m^3^/day. The study includes anaerobic-MFC and aerobic-MFC integrated systems. Microbial electrode jacket dish (MEJ-dish) with hybrid dimension (HD) was invented, first time to authors’ knowledge, to boost anode biofilm growth. The treatment system with MEJ+ (FAB) and MEJ− (MFC) anode are called FAB–MFC and MFC, respectively.

**Results:**

Fragmented variable anode biofilm thickness was observed in FAB than MFC. The FAB–MFC (FAB+) simple technique increases the anode biofilm thickness by ~ 5 times MFC. Due to HD the anode biofilm was fragmented in FAB+ system than MFC. At the end of each treatment cycle, voltage drops. All FAB+ integrated systems reduced voltage drop relative to MFC. FAB reduces voltage drops better than MFC in anaerobic-MFC from 6 to 20 mV and aerobic-MFC from 35–47 mV at 1 kΩ external load. The highest power density was achieved by FAB in anaerobic-MFC (FAB = 104 mW/m^2^, MFC = 98 mW/m^2^) and aerobic-MFC integrated system (FAB = 59 mW/m^2^, MFC = 42 mW/m^2^).

**Conclusions:**

The ∆COD and CE between FAB and MFC could not be concluded because both setups were inserted in the same reactor. The integrated system COD removal (78–97%) was higher than the solitary MFC treatment (68–78%). This study findings support the FAB+ integrated system could be applied for real applications and improve performance. However, it might depend on influent COD, the microbial nature, and ∆COD in FAB+ and MFC, which requires further study.

**Graphic abstract:**

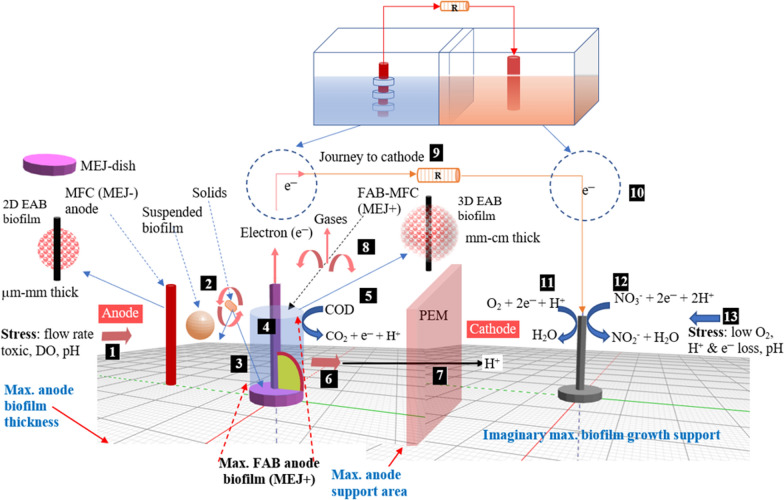

**Supplementary Information:**

The online version contains supplementary material available at 10.1186/s40643-021-00442-x.

## Introduction

The modern wastewater treatment (WWT) system is expected to be energy-autonomous and zero liquid discharge (Li et al. 2013; Stoll et al. 2018). In conventional activated sludge WWT, sludge management accounts for 30–50% of operating costs and the remaining 50% for aeration (Abbassi et al. 2020; Stoll et al. 2018). The aeration demands 0.5–0.29 kWh/m^3^ (3–5% of developed countries national energy budget), which costs $0.12/m^3^ (per annum $7.5 and $0.51 billion in the United States and South Korea, in turn) (Li et al. 2013; Yu et al. 2012), and 0.13–0.14 tCO_2_/m^3^ released (Goto and Yoshida 2019). During treatment, per 1 kg COD removal, ~ 1 kWh consumed (Ahn and Logan 2010) and 0.4 kg (40% of removal) converted to dry solid sludge (Ng et al. 2006). The sludge contains high (66%) organic matter that requires additional treatment (He et al. 2017). Meanwhile, the wastewater is endowed with organic matter that can generate 9.3 times the energy required for treatment (Logan and Regan 2006), equivalent to 4.9–7.9 kWh/kg COD or 7.6 kJ/L that vary based on source (Heidrich et al. 2011). Hence, developing a WWT system that reduces pillar challenges, sludge and aeration, sound sustainable.

Microbial fuel cell (MFC) is a promising sustainable technology with multiple applications such as renewable energy source, WWT, biosensors, and bio-hydrogen production (He et al. 2017). MFCs reduce sludge by 6–11% (Gajaraj and Hu 2014) and 65–71% (Brown et al. 2015) than membrane bioreactor (MBR) and activated sludge treatment, respectively. It is aeration-free treatment technology with a low carbon footprint that generates energy (1.43 kWh/m^3^) and income ($15/kW) (Munoz-Cupa et al. 2021). A typical MFC consists of microorganisms, the substrate as a fuel, electrodes (anode and cathode) separated with a proton exchange membrane (PEM). MFC operated either with mixed culture or monoculture (*Geobacter, Shewanella* sp.) (Logan 2008). These microbes are known as exoelectrogen, oxidizing organic matter and directly transfer the electron to the electrode or with mediators (Nosek et al. 2020). Hence, electrodes are MFC central focuses that attract a critical concern for realizing the practicality (Wei et al. 2011).

However, MFC is currently facing several technical and practical issues for scale-up and commercialization. Several articles were published on the MFC advantages, limitations, and future outlooks (Choudhury et al. 2017; He et al. 2017; Li et al. 2013; Logan and Regan 2006; Santoro et al. 2017; Xu et al. 2016). These authors noted the MFC challenges are related to electrode design, high operating cost, PEM, reactor configuration, and a lack of clear understanding of the electro-biochemical activity. Lessons learned from past studies show that MFC efficiency depends on thick anode biofilm growth and development (Liu et al. 2004; Logan 2008; Logan and Regan 2006). Most studies focus on reactor configuration and optimization, whereas the future of MFC requires a novel electrode to intensify anode EAB growth (Choudhury et al. 2017). Nosek et al. (2020) emphasize that sustainable MFC should modify the existing anode materials for enhanced bacterial attachment and adhesion. Generally, authors who studied anode modification agree that power production increases with increasing anode surface area (Nosek et al. 2020; Zhou et al. 2012). As a result, increasing the bio-electrode surface area becomes the MFC research spotlight.

The core MFC challenge is increasing anode surface area (Lovley 2006). The present means to increase anode surface area were anode modification by nanomaterials, heat, and chemical treatment (Wei et al. 2011). Etching the anode with chemical form groves that increases surface area for microbial attachment (Nosek et al. 2020). For instance, anode treated by ammonium nitrate and nitric acid increased power from the initial 552 mW/m^2^ by 33% (736 mW/m^2^) and 43% (792 mW/m^2^), respectively (Zhou et al. 2012). Previously different anode geometries were studied, such as reticulate vitreous carbon, granular activated carbon, carbon foam, graphite brush electrodes, 3D, graphite pellets, carbon felt, and from flat sheet to packed bed (Yu et al. 2017; Zhou et al. 2012). Up to date, the research gaps on anode modifications were electron loss due to current and mass distribution (Di Lorenzo et al. 2010), complex to prepare or scale-up (Sayed et al. 2020; Xu et al. 2016), expensive (Zhu et al. 2011), and not flexible to manage the biofilm thickness.

The advent of nanotechnology and change from 2D to 3D (three-dimension) electrode results in 3D EAB formation, and it is expected to step MFC forward (Yu et al. 2017). Self-assembled hybrid biofilms (SAHB) can develop a centimeters thick biofilm, a 100× natural biofilm on a planar 2D surface. Nakamura et al. (2009) added α-Fe_2_O_3_ into *Shewanella*, a light-induced α-Fe_2_O_3_/bacteria hybrid network, and increased electroactivity 300× MFC. Chen et al. (2015) developed a porous carbon electrode with a defined pore size (400 nm) by etching SiO_2_ template with sucrose and H_2_SO_4_ in 10% HF and improved power density to 1.6 W/m^2^, ~ 4× carbon felt. The limitation of nanomaterial self-assembly on a 2D electrode was a narrow pore size could not facilitate biofilm growth that requires no < 100 μm (Yu et al. 2017). Also, the fixed pore size hinders the flexible adjustment of the biofilm thickness. It might not be easy to regenerate the electrode (nanosized pore) via simple cleanup (rinsing) for long-term operation. Carbon cloth electroplated with iron nanostructure produces a power output of 80 mW/m^2^, more than 2× bare MFC (Sayed et al. 2020). Still, the long-term 3D electrode (carbon foam) operation is difficult due to anode fouling (Ahn and Logan 2010). In summary, a simple technique to modify anode surface area with flexible mechanisms to increase EAB growth, easily manage the biofilm, and regenerate the electrode is required.

Another best approach to mitigate these problems was integrating MFC with other WWT processes to improve the performance (Xu et al. 2016). The integrated systems enhance pollutant removal efficiency and electricity generation (Abbassi et al. 2020). Previous studies on aerobic or anaerobic pretreatment for MFC-integrated system show reduced energy requirement and the discharge effluent’s COD load (Goto and Yoshida 2019). Wang et al. (2012) developed a novel MBBR-MFC and achieved a higher power density of 6.0 W/m^3^ with an average current of 1.9 ± 0.4 mA. Despite intensive works to improve the MFC-integrated WWT system, there are still crucial research gaps (Chen et al. 2019). These drawbacks were lower electricity generation, controlling the substrate or DO shock, electrode surface area, terminal e^−^ acceptor at the cathode, reactor configuration, voltage drop, and increased internal resistance (Abbassi et al. 2020). Hence, integrating MFC with other WWT systems is not simply connecting and increasing the reactor number and size (Logan 2008); instead, these challenges require a novel approach (Abbassi et al. 2020; Li et al. 2013; Oh and Logan 2007). The substrate shock usually results in voltage drop or unequal electrode potential that lowers electricity generation or the system’s failure (He et al. 2017). Developing a technique to control voltage drop is a fundamental concern for the long-term MFCs operation (Oh and Logan 2007). The future advanced MFCs focus on the MFC-integrated WWT system to increase energy generation through minimizing the internal resistance, increasing EAB thickness through novel design, and MFCs configuration (Abbassi et al. 2020).

To the best of the authors’ knowledge, for the first time, a fragmented anode biofilm (FAB) approach to support variable anode biofilm thickness formation with a flexible controlling technique using MEJ-dish (flexible hybrid 3D electrode) was introduced in this study. It is flexible because when the MEJ-dish is removed easily transforms 3D into a 2D electrode. The MEJ+  is a hybrid dimension (HD) electrode: it comprises 2D and 3D in a single design (Additional file 1: Fig. S8), due to the HD biofilms fragment across the electrode. The study hypothesizes that biofilm growth varies on the 2D (MEJ− = thin) and 3D (MEJ+  = thick) surfaces. The study objectives were to (i) employ comparative research on the anaerobic-MFC and aerobic-MFC integrated system with a novel FAB technique; (ii) examine the effect of increased anode surface area on anode biofilm and voltage drop using domestic wastewater. The performance was evaluated in terms of bioelectrochemical output and COD removal efficiency.

## Materials and methods

### Experimental setup

**Fig. 1:**
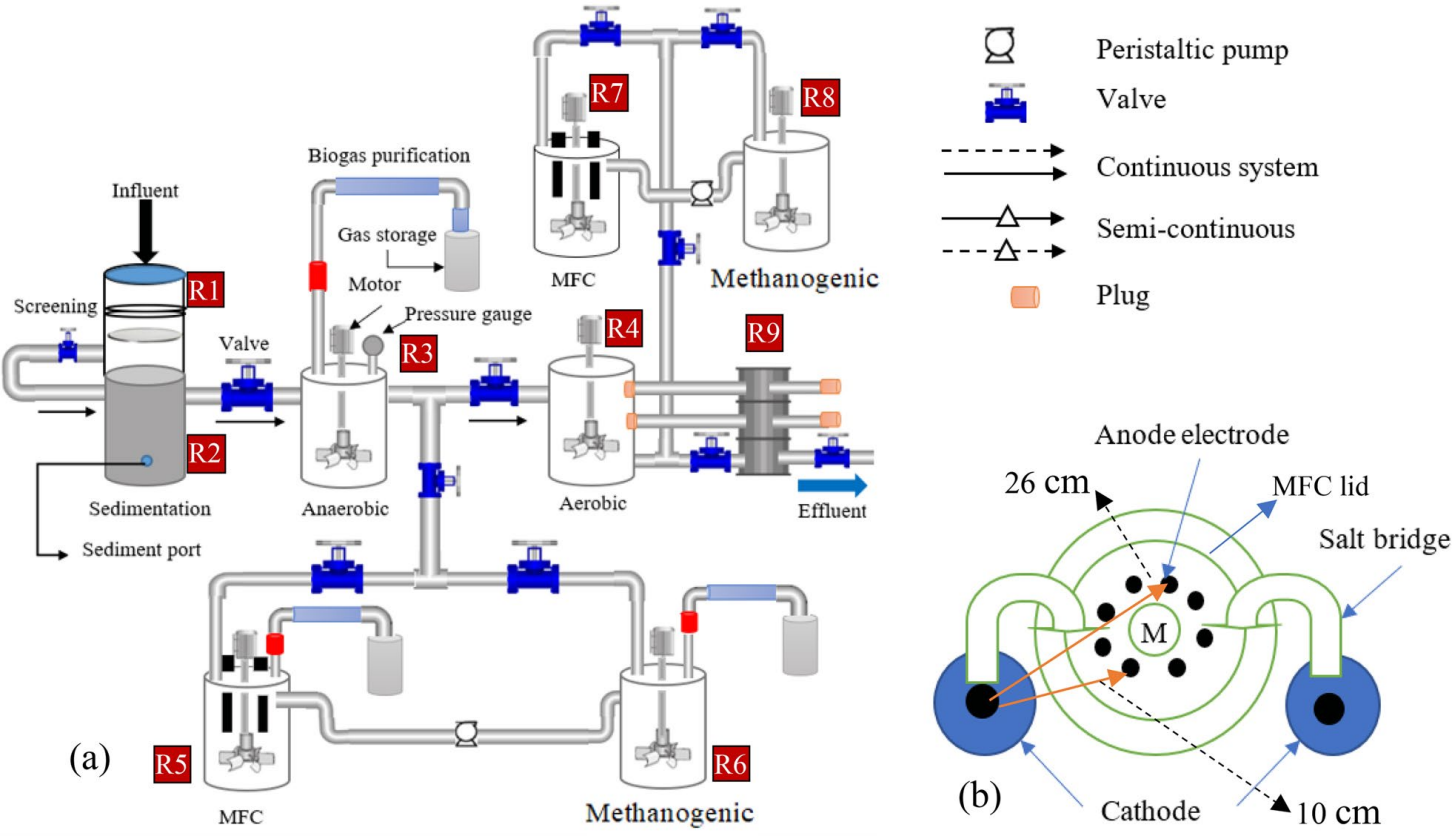
Schematic diagram of **a** the integrated aerobic-MFC and anaerobic-MFC system, and **b** electrode arrangement, not drawn to scale. *M*  motor, *R*  reactor, *R1*  filtration, *R2*  sedimentation, *R3*  anaerobic, *R4 *aerobic (MBBR), *R5* MFC, *R6 *methanogenic, *R7*  MFC, *R8*  methanogenic, and *R9 *adsorpton column. The cathode section is not shown to simplify the diagram

Figure 1 shows the bench-scale anaerobic-MFC and aerobic-MFC integrated system experimental setup. It consists of screening, sedimentation, anaerobic, aerobic, methanogenic, and MFC reactor. The aerobic reactor was designed as a moving bed biofilm reactor (MBBR) with a K3 filter (Ø25 mm × 10 mm) media carrier (Cz Garden Supply, USA); detailed design is shown in Additional file 1: Figs. S3–S4. The reactors were constructed from polypropylene containers, each having a total working volume of 4 L (Ø18 cm × 20 cm in height). The methanogenic and cathode reactors were made from a glass bottle (Ø12 cm and 23 cm high) and a Schott Duran bottle with a working volume of 4 and 1 L, in turn.

**Fig. 2:**
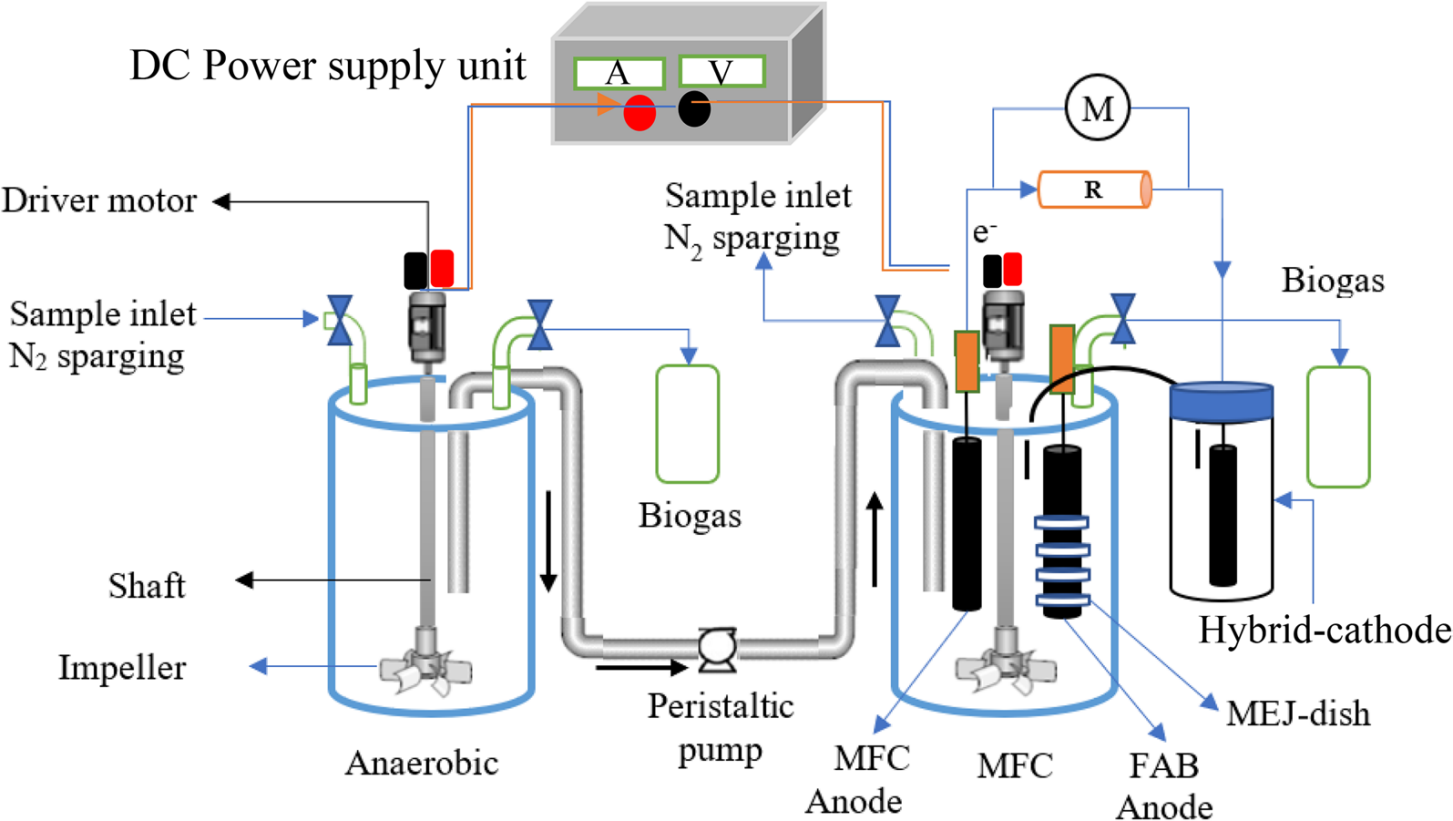
The schematic diagram for a semi-continuous anaerobic-microbial fuel cell (anaerobic-MFC) integrated treatment system. The second air–cathode chamber was not shown to simplify the diagram. *M* a multimeter, *FAB* fragmented anode biofilm, *MEJ-dish* a microbial electrode jacket dish. Arrows indicate the wastewater flow path

**Fig. 3:**
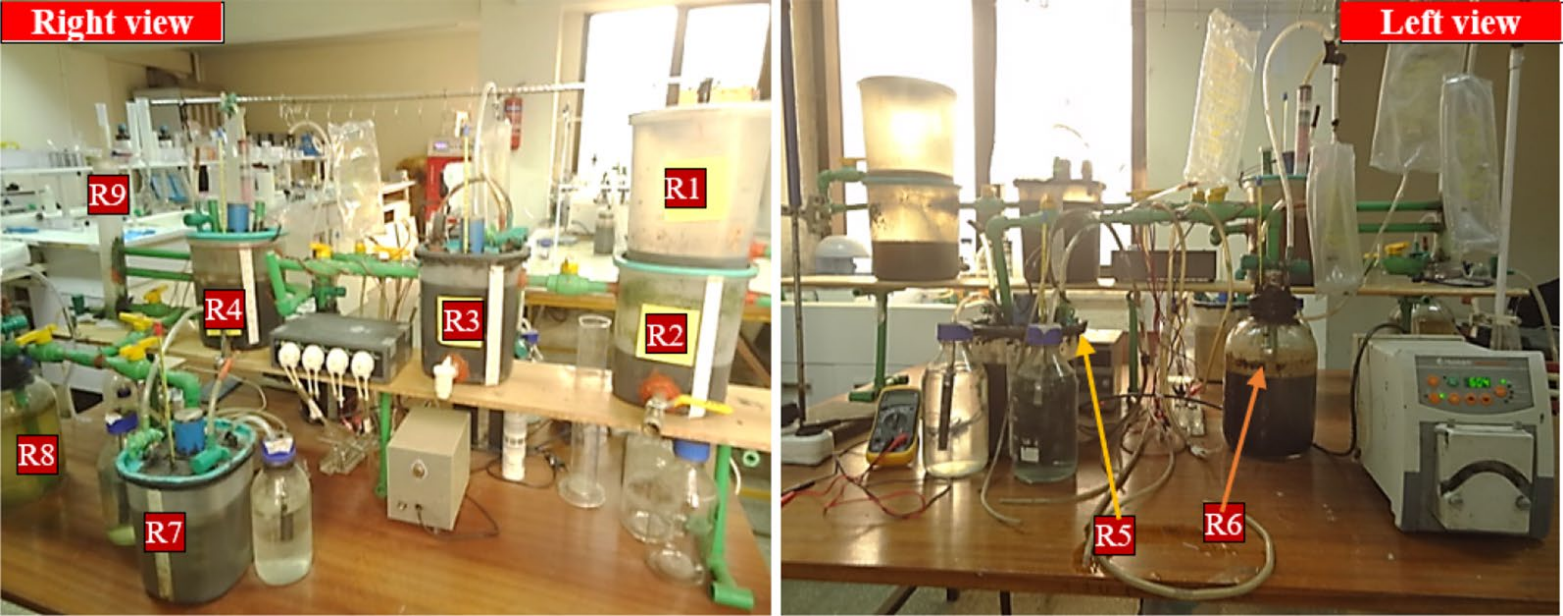
Photo of the constructed MFC-integrated wastewater treatment system, right and left side view. R1–9 indicates the reactor number (for details, see Fig. 1)

The MFCs were double chambered. In the anode chamber, the electrode with microbial electrode jacket dish (MEJ-dish) was considered as FAB (MEJ+), and without MEJ-dish (MEJ−) was labeled as MFC. The MFC treatment system of MEJ+ and MEJ− are called FAB–MFC (FAB+) and MFC, in turn. In this study, both MEJ+ and MEJ− electrodes were inserted into the same MFC anode chamber (Fig. 2). Four MEJ dishes were inserted per electrode (Fig. 3). The MEJ dishes were made from K3 filter media, drilled at the center. The anode reactor consists of eight graphite rods (4 MEJ+, 4MEJ−) arranged concentrically (Fig. 1b), and each anode 2 cm space-separated; this distance was reported to reduce internal resistance (Logan 2008). The electrodes were placed in a sequence (i.e., if first MEJ+ then MEJ−) to minimize the effect of near anodic pH and fuel homogeneity variation.

Two separate hybrid-cathode chambers were placed opposite and an anode in the middle (Fig. 1b). The nearest distance from the hybrid-cathode to the anode was 10 cm, whereas 26 cm (to another anode edge). A hybrid-cathode was made from a single graphite rod (3.5 cm exposed to air and 9 cm immersed in the tap water). The hybrid-cathode was used to avoid the aeration demand; hence, all cathode chambers were not aerated throughout the study period. Each anode is connected to the cathode with an individual circuit. The MEJ+ and MEJ− anodes were connected to a separate cathode chamber with a similar graphite rod. All electrodes were placed perpendicular to the bottom of the reactor (Fig. 2). The MFC electrode arrangement and reactor configuration were similar regardless of the integration mechanism.

Each graphite rod’s total surface area was 40.8 cm^2^ (Ø1 cm × 12.5 cm in height) without including the pores formed during abrasion with sandpaper. A single K3 MEJ-dish (deduct anode diameter, Ø15 mm × 10 mm) provides an additional 1.8 cm^2^. A total of 4 MEJ-dishes increased the top surface area by 7 cm^2^ to support microbial attachment on the anode surface. Hence, assuming the MEJ-dish was conductor, and the junction cover between the anode and MEJ-dish was negligible, it increased the anode’s total surface area by 33 cm^2^ (81%).

## Operation

**Fig. 4:**
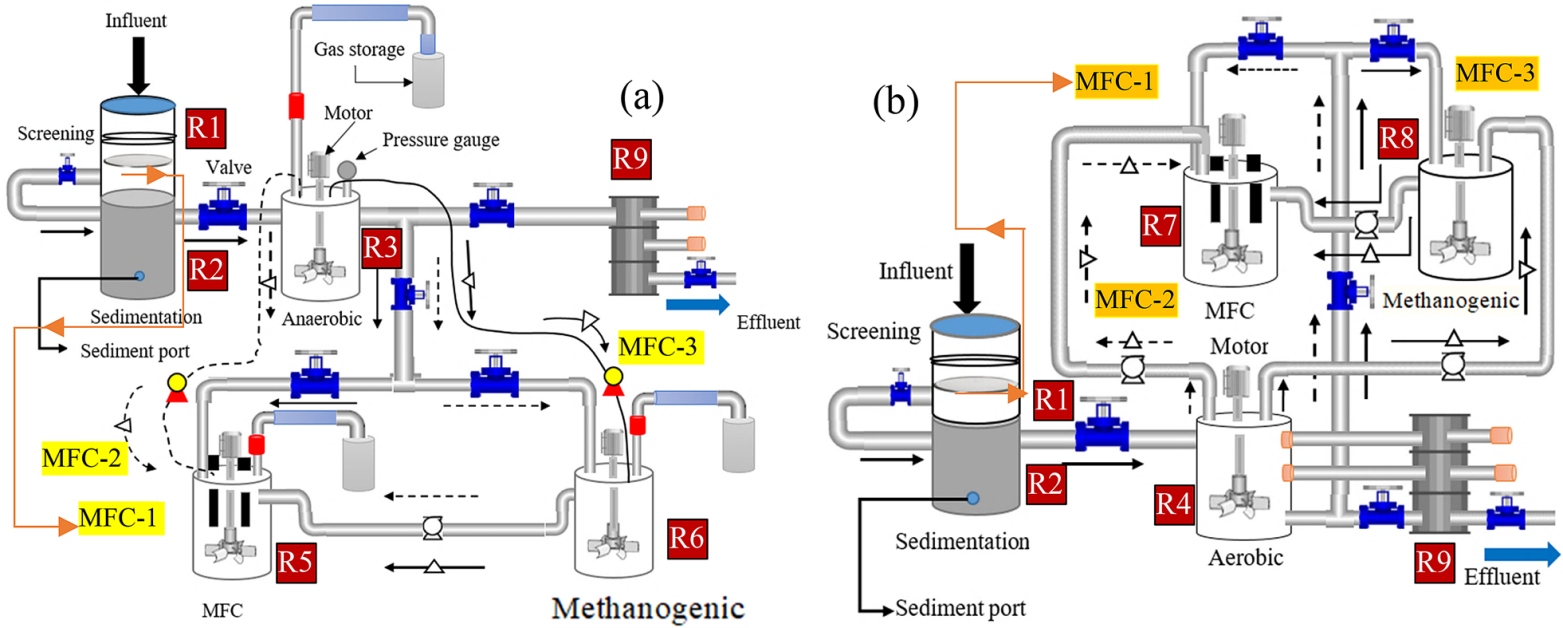
Operation mechanisms for **a** anaerobic-MFC and **b** aerobic-MFC system. Within each system, MFC-1 is considered as a direct-fed MFC (control) or standalone system, whereas the MFC-2 and -3 are integrated systems. The arrows indicate the water flow path

Anaerobic-MFC and aerobic-MFC integrated system operation is shown in Fig. 4a, b. A similar flow pattern was followed among the aerobic or anaerobic-MFC integrated systems. The study was divided into three phases: the first phase (MFC-1), the influent was directly fed into the MFC. In the second phase (MFC-2), hydrolysis (anaerobic) effluent was fed to FAB–MFC. In the third phase (MFC-3), the anaerobic effluent was fed to the methanogenic reactor then transferred to FAB–MFC. The reactors were operated in fed-batch mode with four stages in 24:1 h sedimentation-R2, 30-min fill, 21 h react (6 h mix), 1 h settle, and 30-min decant. Equal 4 L/day WW were decanted and filled. The integrated systems were compared under different pretreatment with similar 1 day of HRT in MFC at 0.76 kg-COD/m^3^/day. The HRTs were 1, 2, and 3 days in MFC-1, -2, and -3, respectively.

The wastewater fill and decant were conducted using a programmable auto-dosing pump (DP-4, Jebao Co., Ltd., China) to minimize the effect of MFC oxygen contamination. An aquarium air pump (SB-9903/A, SOBO^®^, China) with a capacity of 5 L/min was used to supply compressed air into the aerobic MBBR reactor intermittently. The liquid contents were mixed using an overhead mounted motor to keep even biomass distribution. Each integrated system was operated for more than 30 days before evaluating the performance. The reactors were inoculated with mixed culture and operated at room temperature (25 ± 1 ℃) without sludge returning, pH adjustment, or nutrient addition. All the systems were considered steady-state when voltage output was less than 0.01 V/h variation under 1000 Ω external load.

### Wastewater sampling and characterization

Wastewater (WW) sampling and analysis were conducted according to APHA (2005). Influent WW used for this study was collected from a primary clarifier of Mickey Leland condominium domestic WWT plant (Asko, AA, Ethiopia). WW was collected every 3 days to expose the designed treatment system to actual COD fluctuation in the primary clarifier. The collected samples were either used immediately upon arrival to the laboratory or stored at 4 ℃ in a refrigerator. Influent and treated water samples were taken per specified HRT and analyzed for COD concentration. The raw WW, inoculant, and inoculum were analyzed for volatile solids, total solids, total alkalinity, ammoniacal nitrogen (${\text{NH}}_{4}^{ + }$-N), and total phosphorus. The temperature was measured using the reactor top-mounted thermometer, while DO and pH were measured using a probe. All physicochemical and bioelectrochemical analyses were conducted at least in duplicate. Table 1 summarizes the raw wastewater, inoculum, and inoculant physicochemical characteristics.

**Table 1:** Characteristics of raw, inoculum, and influent wastewater (mean ± SD)

Parameters	Raw wastewater	Inoculant	Inoculum
pH (−)	7.51 ± 0.07	6.43 ± 0.06	6.97 ± 0.03
Total alkalinity (mg/L as CaCO_3_)	35 ± 21	144 ± 15	130 ± 42
Total solid (mg/L)	158 ± 29	3277 ± 416	2894 ± 83
Volatile solid (mg/L)	366 ± 59	1559 ± 211	1347 ± 60
Total COD (mg/L)	755 ± 160	9760 ± 551	5782 ± 50
Ammonia-N (mg/L)	35 ± 9	272 ± 41	168 ± 21
Total phosphorus (mg/L)	9 ± 3	31 ± 11	17 ± 5

### Anode biofilm sampling and characterization

Millo (2015) and Bakke et al. (2001) optical methods were adopted with modification to measure the anode biofilm thickness (BT). The anode electrodes were scrapped using a scalpel until the graphite electrode surface was observed, the biofilm tip was fixed facing the optical microscope objective. The biofilms were dispersed on the electrode surface; at least five *n* independent biofilms (5 ≤ *n* ≤ 10) per 1 cm of anode projected area (4.71 cm^2^) were measured. It was difficult to quantify all BT at different biofilm growth stages, from prokaryotic cells to matured biofilms. Hence, the top BT at the specified *n* range was reported, excluding outliers or infrequently observed BT (did not occur at least five times per cm of electrode). The method was validated using a standard, Kapton^®^ tape (DuPont Co., USA) with a manufacturer-reported thickness of 88.9 μm, including the glue part. A tape was wrapped around a bare electrode without overlapping. The measurement of the tape thickness was carried out as described above. The test was run in triplicate (*n* = 5), and BT measurement accuracy was about 95% (84 ± 1.4 μm) with ± 6 μm precision (93%).

### Electrochemical analysis

The anode and cathode were connected across 1000 Ω external resistance (*R*_ext_) unless otherwise stated. The voltage was measured using a digital multimeter (XL830L, China). Data were recorded at least three times per day, and the daily average was presented. The current (*I*) was calculated based on Ohm’s law as *I* = *V*/*R*_ext_ and power output (*P*) = *IV* (*V*^2^/*R*_ext_), where *I* is the current (A), *V* is the voltage (V), and *R*_ext_ is the external resistance (Ω). Power density (mW/m^2^) = *P*/*A*, and current density (mA/m^2^) = *I*/*A*, where *A* is normalized to the submerged cathode area (*A* = 29.83 cm^2^) due to variation in anode chamber (MEJ+ or MEJ−), and others as described previously.

### Calculations

The COD removal efficiency and HRT were calculated using Eqs. (1) and (2), respectively.

1 $${\text{COD}}_{\text{RE}}\text{ = }\left(\text{1} - \frac{{\text{COD}}_{\text{EF}}}{{\text{COD}}_{\text{IN}}}\right) \times 100\%,$$

where COD_RE_ is the COD removal efficiency (%), COD_IN_ is an influent COD concentration (mg/L), and COD_EF_ is an effluent COD concentration (mg/L).

2 $$\text{HRT} = \frac{{{V}}_{\text{reactor}}}{{Q}},$$

where *V* is the volume of the reactor (m^3^), and *Q* is the influent flow rate within a specified time (m^3^/day).

Coulombic efficiency (CE) is the ratio of total charge obtained practically (*C*_P_) to the theoretical value (*C*_T_) obtained from complete substrate oxidization. CE was calculated based on Eq. (3) (Wen et al. 2009):

3 $${\text{CE}} = \frac{{{C}}_{\text{P}}}{{{C}}_{\text{T}}} \times 100\% = \frac{{8}{\int }_{0}^{{\text{t}}_{\text{b}}}{I {\text d}t}}{{\text{F}}{\text{V}}_{\text{an}} \Delta {\rm COD}} \times 100\% = \frac{{8} \, \text{I t}}{{\text{F}}{\text{V}}_{\text{an}} \Delta {\rm COD}} \times 100\%,$$

where *F* is Faraday’s constant (96,485 C/mol), ΔCOD is the difference between the influent and effluent COD (mg/L) over a time (*t*_b_), *I* is the current (A), and *V*_an_ is the liquid volume in the anode (L).

### Data analysis

The collected data were analyzed using IBM SPSS Statistics for Windows, version 20 (IBM Corp., NY, USA). One-way analysis of variance (ANOVA) was used to compare the MFC bioelectricity or treatment system performance, and *p* < 0.05 was considered a significant variation.

## Results and discussion

### Performance of anaerobic-MFC

**Fig. 5:**
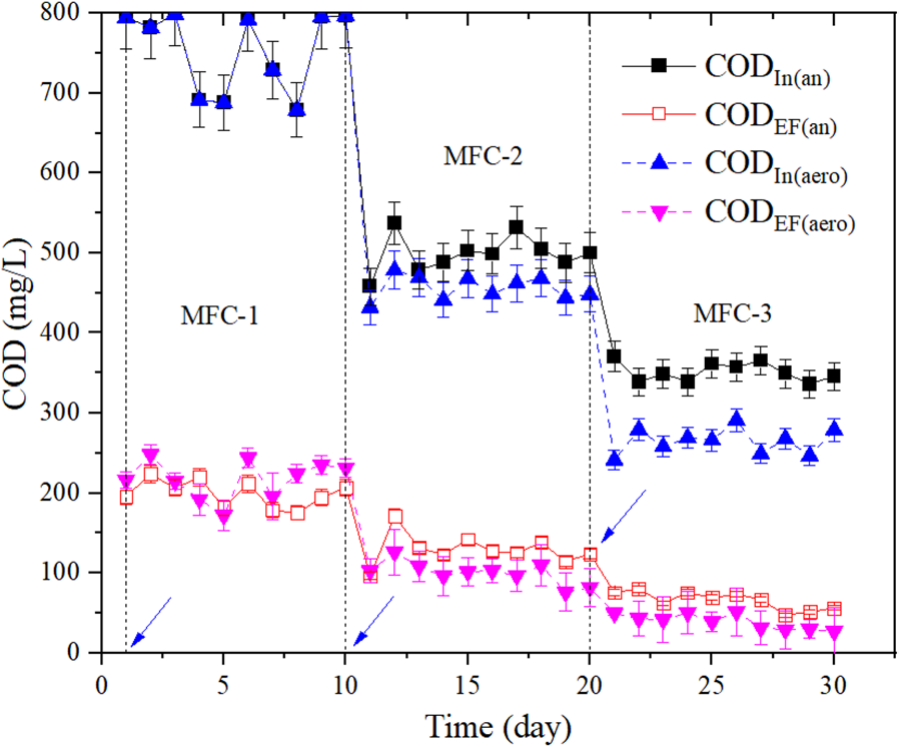
COD concentration in the influent (COD_IN_) and effluent (COD_EF_) for aerobic-MFC and anaerobic-MFC integrated system. (an) indicates anaerobic-MFC, and (aero) shows an aerobic-MFC integrated system. The arrows show treatment phase change: wastewater replacement and the systems were allowed to stabilize again. The pre-stabilization period was not shown in the graph. Error bars indicate standard deviation

The anaerobic-MFC integrated treatment system COD concentration in the influent (COD_IN_) and effluent (COD_EF_) is shown in Fig. 5. The MFC-1, -2, and -3 COD_IN_ were on average 755, 496, and 351 mg/L, respectively. The anaerobic-MFC integrated COD_IN_ (SD = 52 mg/L) was relatively more stable than directly fed MFC (SD = 23 mg/L). The COD_EF_ decreases as the treatment steps increases, MFC-1 > -2 > -3. Despite COD_IN_ fluctuations, the COD_EF_ stabilized as the treatment period was extended in all the treatment setups. This result suggests that the COD_EF_ was more affected by the operation period than the observed COD_IN_ variation. This stability might be associated with the microbial establishment as the operation period (reactor age) increases within 1 day of HRT.

**Fig. 6:**
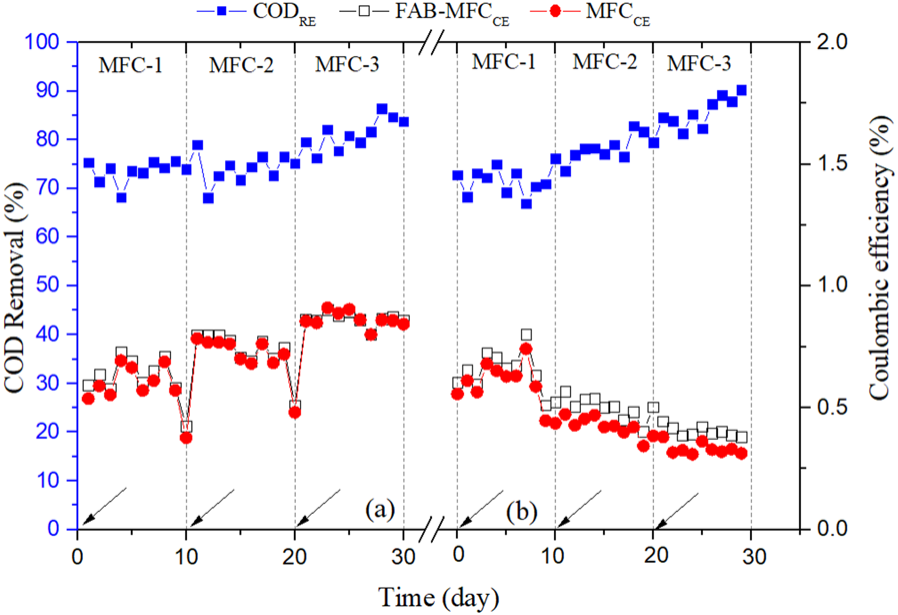
Effects of **a** anaerobic-MFC and** b** aerobic-MFC integrated system on the FAB–MFC performance in terms of COD removal efficiency (COD_RE_) and coulombic efficiency. FAB–MFC_CE_ and MFC_CE_ show FAB–MFC and MFC coulombic efficiency, respectively. The arrows indicate treatment phase change: previous phase contents emptied, and the system allowed to re-stabilize

In standalone MFC, the COD removal efficiency (COD_RE_) was in the range of 72–78%, whereas 78–94% in the anaerobic-MFC integrated system at a steady state (Fig. 6). There was a significant COD_RE_ difference between the standalone MFC and anaerobic-MFC integrated systems. In the MFC, the COD_IN_ is not solely used for electricity generation, but also consumed by non-EAB, sediment in the sludge, cell growth, untapped during treatment, and remain effluent (Liu et al. 2004). Even the COD might be used to generate electron (e^−^) but lost due to bioelectrochemical limitations.

**Fig. 7:**
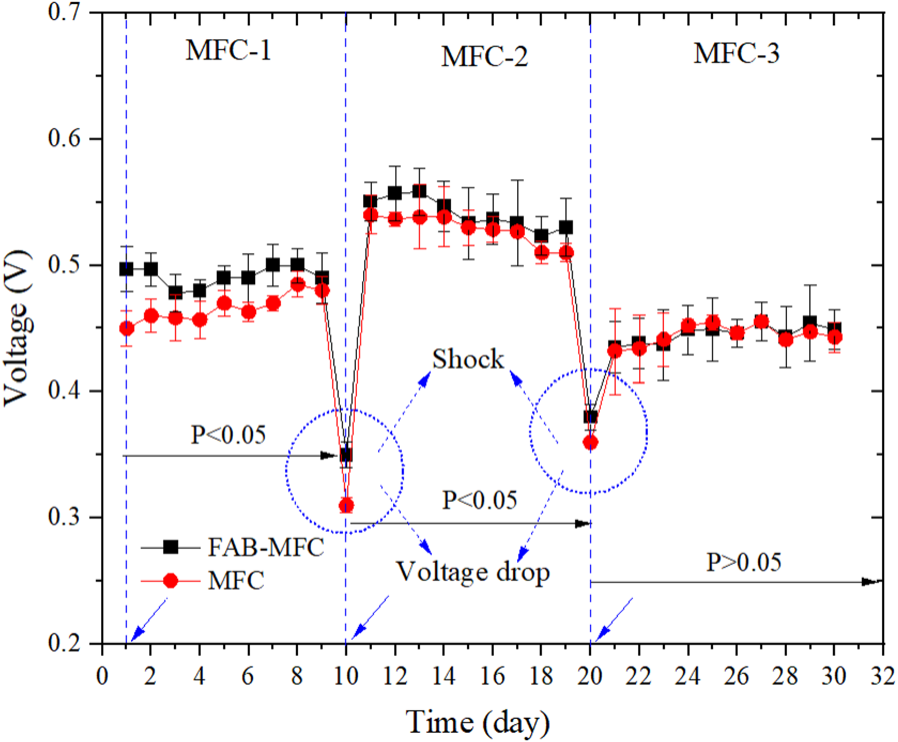
The voltage generated in the lab-scale anaerobic**-**MFC integrated domestic wastewater treatment system. Error bars indicate the standard deviation. The *p *values indicate the significant difference (*p* < 0.05) between the FAB–MFC (FAB+) and MFC. External load 1000 Ω. Shock means the point where the minimum voltage was observed during the transition to the next treatment phase. A circle with arrows indicates wastewater replacement and system shock

In the anaerobic-MFC integrated system, the mean voltage generated from the higher to lower was MFC-2 > -1 > -3 (Fig. 7). The figure shows that the highest voltage in MFC-2 was 0.56 (FAB) and 0.54 V (MFC), which was 0.11–0.13 V higher than the lowest values observed in MFC-3. Likewise, in MFC-2, the maximum power density (MPD) of 104 mW/m^2^ (FAB) and 98 mW/m^2^ (MFC) were observed at the highest current density of 187 mA/m^2^ (FAB) and 181 mA/m^2^ (MFC), which was lower than 264 mW/m^2^ (Wen et al. 2009), and higher than 37 mW/m^2^ (Zhu et al. 2011) and 96 mW/m^2^ (Chen et al. 2019). The MPD of the MFC-1 and -3 were 84 and 70 mW/m^2^ for FAB, 79 and 70 mW/m^2^ for MFC, respectively (Table 2).

**Table 2:** Comparisons of FAB–MFC integrated system with other studies

WW type	COD_IN_ (COD_EF_) COD_RE_	MFC type (PEM) HRT	Max. P (I) CE	References
Standalone MFC				
Glucose	1200 (24) 98%	S-MFC (Nafion) –	262 (−) 55%	Liu and Logan (2004)
Synthetic	1000 (310) 69%	S-MFC (Nafion) –	81 (−) 30%	Di Lorenzo et al. (2010)
Swine	6500 (3400) 49%	S-MFC (PTFE) 3 days	0.15 (−) 1.0–15%	Goto and Yoshida (2019)
Swine	7500 (3075) 22%	S-MFC (No PEM) 0.7 days	750 (1) 23%	Kim et al. (2016)
Brewery	627 (366) 42%	S-MFC (Nafion) 2.13 h	264 (1.79) 20%	Wen et al. (2009)
Domestic	400 (140) 65%	S-MFC (No PEM) 8.8 h	300 (−) 36%	Kim et al. (2015)
Domestic	220 (44) 80%	S-MFC (Nafion) 3–33 h	26 (−) 3–12%	Liu et al. (2004)
Domestic	– (–) –	D-MFC (Salt bridge) –	0.3 (18) 19%	Min et al. (2005)
Domestic	375 (150) 60%	D-MFC (Salt bridge) –	25 (100) 0.25%	Rodrigo et al. (2007)
Domestic	820 (180) 78%	D-MFC (Nafion) 14 days	817 (0.55) 32%	Bose et al. (2018)
Domestic	300 (135) 55%	S-MFC (Nafion) 78 h	28 (85) 28	Liu and Logan (2004)
Domestic	108 (37) 66%	SE-MFC (Fabric) 6 h	149 (250) 6%	Yu et al. (2012)
Domestic	500 (371) 26%	S-MFC (No PEM) 0.2 h	422 (15) 0.7%	Ahn and Logan (2010)
Domestic	299 (87) 71%	S-MFC (No PEM) 2 h	103 (420) 18%	You et al. (2006)
Domestic	600 (174) 71%	D-MFC (CEM) 0.69 d	180 (−) –	Ye et al. (2019)
Domestic Domestic	755 (200) 74%	MFC-1^An^ (Salt bridge) 1 day FAB-1^An^ (Salt bridge) 1 day	79 (163) 0.73% 84 (168) 0.69%	This study This study
Domestic Domestic	755 (218) 71%	MFC-1^A^ (Salt bridge) 1 day FAB-1^A^ (Salt bridge) 1 day	78 (161) 0.80% 87 (171) 0.74%	This study This study
MFC-integrated system				
Brewery	708 (103) 80%	IVCW-MFC (No PEM) 2 d	− (50) 0.39%	Liu et al. (2019)
Synthetic	1500 (375) 75%	VCW-MFC (No PEM) 4 d	16 (70) 0.15%	Yadav et al. (2012)
Pulp	4500 (2970) 66%	MBBR-MFC (Nafion) 3 days	96 (185) 0.62%	Chen et al. (2019)
Domestic	537 (17) 97%	MBR-MFC (No PEM) 1 day	− (−) 0.05%	Gajaraj and Hu (2014)
Domestic	430 (7) 92%	MFC-MBR (No PEM) 5 days	51 (0.2) 5.9%	Su et al. (2013)
Synthetic	600 (60) 90%	MBR-MFC (No PEM) 2 h	140 (0.52) 7%	(Li et al. 2015)
Domestic	1080 (32) 97%	MFC-MBR (No PEM) 3 h	380 (2000)8.5%	Malaeb et al. (2013)
Domestic Domestic	755 (129) 83%	An-MFC-2 (Salt bridge) 2 days An-FAB-2 (Salt bridge) 2 days	98 (181) 0.80% 104 (187) 0.79%	This study This study
Domestic Domestic	755 (66) 91%	An-MFC-3 (Salt bridge) 3 days An-FAB-3 (Salt bridge) 3 days	70 (153) 0.90% 70 (153) 0.91%	This study This study
Domestic Domestic	755 (100) 87%	A-MFC-2 (Salt bridge) 2 days A-FAB-2 (Salt bridge) 2 days	42 (119) 0.57% 59 (140) 0.47%	This study This study
Domestic Domestic	755 (39) 95%	A-MFC-3 (Salt bridge) 3 days A-FAB-3 (Salt bridge) 3 days	12 (63) 0.50% 18 (78) 0.39%	This study This study

*WW* wastewater, *COD*_*IN*_* (COD*_*EF*_*) COD*_*RE*_ COD influent (mg/L), effluent (mg/L) and removal efficiency (%), respectively, *Max. P (I) CE* maximum power density as mW/m^2^ (current density, mA/m^2^) coulombic efficiency (%)

*S-MFC* single chamber MFC, *D-MFC* double chambered MFC, *SE-MFC* submerged-exchangeable-MFC, *MFC-1*^*An*^ anaerobic-D-MFC-1, *FAB-1*^*An*^ anaerobic-D-FAB–MFC-1, *MFC-1*^*A*^ aerobic-MFC-1, *FAB-1*^*A*^ aerobic-D-FAB–MFC-1

Bar shows not reported or could not be calculated from the given information; d shows day, h for an hour

*PTFE* polytetrafluoroethylene, *IVCW-MFC *integrated vertical flow constructed wetland-MFC, *PEM* proton exchange membrane, *CEM *cation; exchange membrane, *MBR* membrane bioreactor, *A-MFC (FAB)* aerobic-MFC (FAB), *An-MFC (FAB)* anaerobic-MFC (FAB), *FAB* fragmented anode biofilm

During the transition from MFC-1 to -2 (or -3), the contents were emptied and replenished with a fresh substrate to reduce the proceeding system interference. As shown in Fig. 7, a voltage generation drop was recorded in FAB–MFC (FAB+) and MFC (0.5 to 0.31 V); this was noted as system shock. However, the FAB+ reduces voltage drop from 6 to 20 mV more than MFC. The voltage drop recorded in this study was within the 0.2 V range, higher than Gajaraj and Hu (2014) findings (0.12 V). Factors affecting MFC performance are external resistor, substrate, DO, pH, and microbial diversity (Lin et al. 2013). Hence, it could be due to the COD_IN_ load variation.

On the other hand, it might arise due to the feeding technique attributed to oxygen contamination and wastewater. The MFC anode reaction products could not be electron alone and include gases such as CO_2_, ${\text{NH}}_{4}^{ + }$-N, CH_4_, and H_2_ (Abbassi et al. 2020; Li et al. 2013; Santoro et al. 2017). The selective COD and ammonia removal are crucial to increase the MFC performance (Lin et al. 2013). Hence, removing the anode byproducts might reduce the competition between exoelectrogens and other anaerobic microbes such as methanogens.

### Performance of aerobic-MFC

Figure 5 shows the COD concentration in the influent and effluent of the aerobic-MFC system. MFC can be operated from 22 to 127,500 mg/L COD (Zhang et al. 2009). The average COD_IN_ observed in MFC-1 (755 mg/L), MFC-2 (456 mg/L), and MFC-3 (265 mg/L) fall within the recommendable MFC COD_IN_ range. Like the anaerobic-MFC, the COD_EF_ declines as the pretreatment level increases: MFC-1 > -2 > -3. The aerobic-MFC integrated system COD_RE_ was (84–97%) better than solitary MFC (68–78%) treatment at a steady state.

The highest COD_RE_ was observed in aerobic-MFC than the anaerobic-MFC system, but lower voltage generation. This contradictory effect indicates the major portion of COD_IN_ might not be used for voltage generation instead consumed by aerobes, or the generated e^─^ was removed via an e^−^ acceptor such as oxygen in the aerobic-MFC system. Li et al. (2013) suggested feeding low-strength wastewater into MFC directly and to apply (anaerobic) pretreatment for high-strength wastewater. Hence, from an energy performance point of view (practical voltage generation), wastewater with ~ 800 mg/L COD_IN_ might be sound if MFC was applied before the aerobic treatment (MFC-aerobic better than aerobic-MFC).

**Fig. 8:**
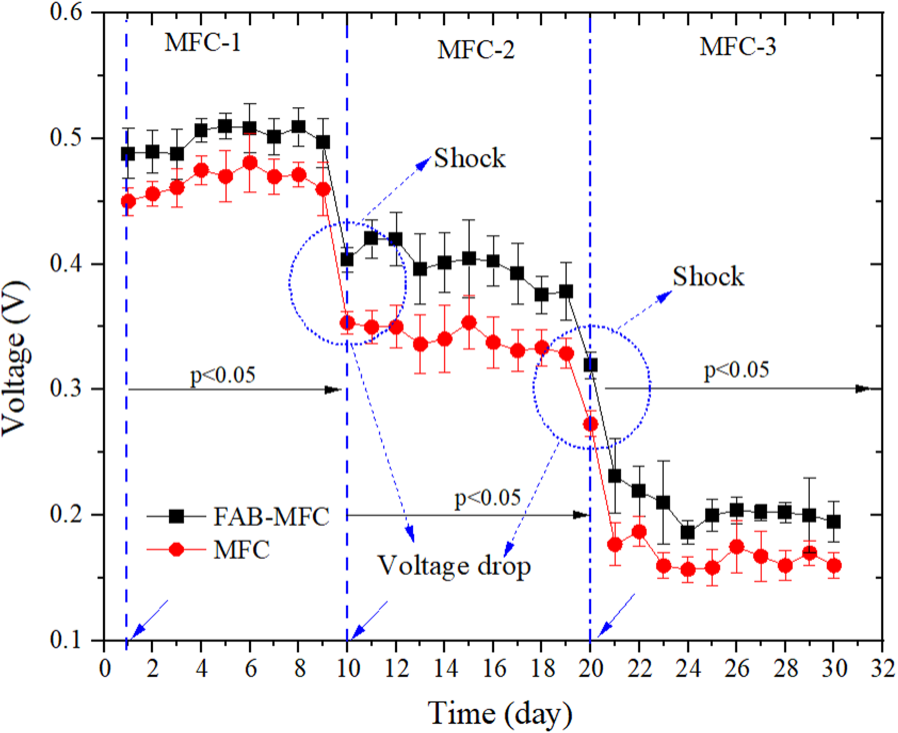
The voltage generated in the aerobic**-**MFC integrated domestic wastewater treatment system. Error bars indicate the standard deviation (SD). External load 1000 Ω. The *p* < 0.05 indicates a significant difference between the FAB–MFC and MFC. A circle with arrows indicates wastewater replacement and system shock

The voltage generation decreases when the aerobic treatment sequence before MFC increases (MFC-1 > -2 > -3). The highest voltage production by the MFC-1, -2 and -3 were 0.51, 0.42, and 0.23 V, respectively (Fig. 8). Inconsistent with the findings, Chen et al. (2019) observed 0.5 V using novel MBR-MFC at a similar 1 kΩ external load. The voltage declines in MFC-2 might be mainly attributed to the aerobic influent wastewater condition and associated COD_IN_ decline, which affects the electroactive microorganisms. These microbes are sensitive to DO, oxidize organic matter under anaerobic conditions, and release electrons to the anode (Logan 2008). However, despite lower DO in the MFC-3 (data not shown), the voltage output decreases due to enhanced COD removal during the pretreatment. Overall, the voltage generation declined as the influent pretreatment increases. In agreement, Zhang et al. (2013) noted integrating MFC with denitrifying system improves nitrate removal, but lowers energy output.

The MFC-1 MPD was higher than MFC-2 and -3. It was 87 and 78 mW/m^2^ in FAB and MFC, respectively, at the maximum current density of 171 mA/m^2^ (FAB) and 161 mA/m^2^ (MFC). These results were in accordance with Yu et al. (2012) that noted MPD of 116–149 mW/m^2^ from low-strength domestic wastewater (COD_IN_ = 100 mg/L), and Chen et al. (2019) studied a novel MBBR–MFC integrated system and reported MPD of 95 mW/m^2^ at 1 day HRT. However, it is lower than 264 mW/m^2^ MPD reported by Wen et al. (2009) while operating brewery wastewater, COD_IN_ = 627 mg/L. The MPD declines in the integrated system of MFC-2 (FAB = 59 mW/m^2^, MFC = 42 mW/m^2^) and MFC-3 (FAB = 18 mW/m^2^, MFC = 12 mW/m^2^) (Table 2). This declining trend could be due to upstream pretreatment that decreased organic content in the MFC-2 and -3 influent. Power density decline with decreased external resistance could be due to limited e^−^ transfer to the cathode at higher external load (Zhu et al. 2011). FAB power output betterment could be due to thick EAB, which may reduce ohmic loss unlike the packed bed granules and ease substrate diffusion into EAB, consequently hampering the DO effect.

In MFC-1, at startup, the FAB–MFC (FAB+) voltage generation was 0.04 V higher than MFC; after 6 days of operation, the gap narrows to 0.03 V (Fig. 8). The EAB requires an optimum of 7 days to grow on the MFC anode surface (Arbianti et al. 2018). FAB (MEJ+) might speed up the EAB colonization and growth, but against this case, all the setups were operated for more than a month to reach a steady state before the experiment startup (variation < 0.01 V/h). Hence, most likely, the thick biofilms formed on the FAB anode withstand aerobic influent shock and stabilize voltage generation. Like the anaerobic-MFC, the aerobic-MFC voltage generation was affected during phase interchange. In the aerobic-MFC integrated system, the FAB+ reduces the voltage drop by 14 mV than MFC.

The MFC-2 MPD difference between FAB+ and MFC in aerobic-MFC (17 mW/m^2^) was ~ 3 times higher than anaerobic-MFC (7 mW/m^2^). Hence, the FAB critical role was revealed when the MFC was exposed to anaerobically treated influent. This finding supports, the thick anode biofilms might be valuable for the EABs’ functional stability during DO intrusion. The observed data suggest an interaction effect between the thick anode biofilm, COD_IN_, EAB, and DO on FAB+ bioelectricity generation and treatment performance.

### Effect of FAB on MFC-integrated system performance

**Fig. 9:**
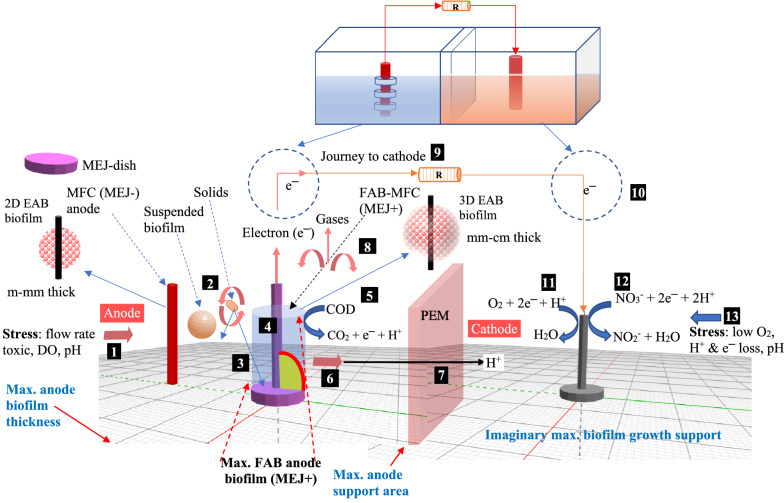
The proposed conceptual scheme of anode biofilm growth on support media (MEJ-dish) against the hypothetical maximum (max.) surface area and biofilm thickness. The anode biofilm thickness was proposed without assuming extended growth. The numbers in a box (1–13) indicate the MFC substrate and electron flow (Additional file 1: Fig. S1). The MEJ-dish supports to form thick (FAB) anode biofilm (No. 3)

**Fig. 10:**
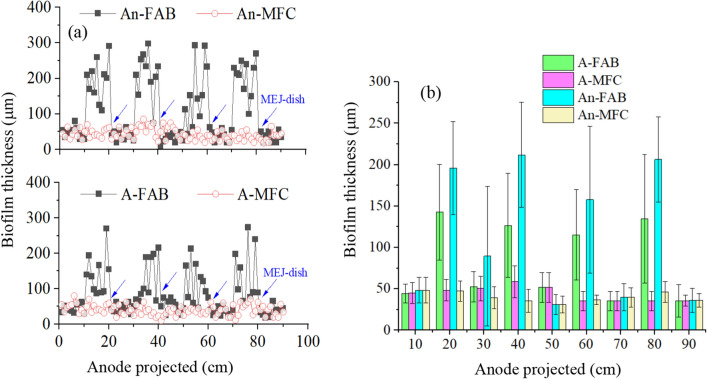
The anode biofilm thickness (BT) profile in the aerobic-MFC (A-FAB and A-MFC) and anaerobic (An-Fab and An-MFC) integrated system. The overall **a **BT distribution and **b** mean ± SD. Each data point indicates the top BT within multiple measurements of *n* independent biofilms (5 ≤ *n* ≤ 10) per 10 mm distance excluding outliers, but not the exact spatial and temporal BT distribution on the electrode. The arrows indicate MEJ-dish inserted location on the anode. *FAB*  fragmented anode biofilm

Figure 9 displays the MEJ+ (FAB) proposed biofilm growth against MEJ− (MFC) anode. The conceptual scheme shows that the MEJ-dish was designed to support EAB growth and increase anode biofilm thickness. As expected, fragmented (thick and thin) anode biofilms were observed in FAB than MFC (Additional file 1: Fig S9). Of course, the biofilm thickness on the MFC anode was heterogeneous and varied across the surface; however, the FAB magnifies this variation into a significant difference (Fig. 10). Similarly, Li et al. (2016) reported thick electrical conductor biofilm. The present study did not determine the biofilm electrical conductance, despite previous studies suggesting centimeter-long biofilm conductivity. The anode biofilm structures are dependent on anode materials (Nevin et al. 2008), more bacterial adhesion linked with more thick biofilm, and lower electrical loss (Nosek et al. 2020). Hence, the FAB peculiar anode biofilm structure could be due to MEJ-dish.

The FAB anode average biofilm thickness was ~ 5 times higher than MFC in anaerobic-MFC (FAB = 188 ± 67 μm, MFC = 43 ± 17 μm) and aerobic-MFC (FAB = 127 ± 62 μm, MFC = 40 ± 14 μm) (Fig. 10). These values were within previously reported average anode biofilm thickness: 66 ± 16 μm (Lee et al. 2009), 42 ± 3 μm (Read et al. 2010), and 150 μm (Millo 2015). *G. sulfurreducens* form EAB that conduct e^−^ about 100 μm thick (Malvankar et al. 2011). However, Li et al. (2016) reported a 1 mm conductor biofilm length (10×) using mixed species. Infrequently, the biofilm on the MEJ-dish reaches a centimeter long (Additional file 1: Fig. S8), which might be due to biofilm detachment. It shows the proposed FAB method could support the EAB life cycle from attachment-to-detachment (genesis-to-end). Due to unknown reasons, the biofilm thickness in anaerobic-MFC was thicker than aerobic-MFC. This thickness variation might be due to lower synergetic association in the aerobic exposed anode. Hence, exposing the anode biofilm to aerobic influent might slice the thickness.

This study was not the pioneer to report a centimeter-long biofilm. Bacteria develop a wide range of biofilm thickness; for example, *Bacillus subtilis* cultured on agar media form 600 μm thick and 1.2 cm long (Wang et al. 2015), 200–500 μm on MBBR filter carrier media (Piculell et al. 2016). Using the SAHB approach, Nakamura et al. (2009) added α-Fe_2_O_3_ into *Shewanella* to develop a thick anode biofilm. But the technical viability to practical application remains a challenge. The FAB presents a simple method by making a micro or macrostructure on the electrode surface to enlengthen the EAB thickness. Unlike so far reported, the FAB surface topography is a removable electrode jacket (coated), which supports the EAB life cycle and easily regenerates the electrodes. However, it was not without demerit that requires optimizing the MEJ-dish type (size) that affects the junction point while socketing with the electrode. It covers the contact surface, creates a blind spot (prevents biofilm growth and e^−^ collection on the surface), and affects power output efficiency. In addition, the pore structure on the top side of K3 filter media probably discourages further biofilm enlargement on MEJ-dish. These MEJ-biofilm-electrical effects could be a subject for future studies and debate.

The FAB thick anode biofilm formation might provide a multilayer advantage. Thick anode biofilm formation and high current production is associated with pili that contribute to the e^−^ transfer to the anode (Nevin et al. 2008) and reduce competition between oxygen and anode e^−^ acceptor (Nosek et al. 2020). The extracellular biofilm heteropolysaccharides and *c*-type cytochrome protein complex transfer e^−^ to the anode (Santoro et al. 2017). Probably, the interdependent function of biofilm materials such as EPS and pili complex could enhance the e^−^ transfer in the FAB–MFC. On the other hand, suspended microbes, including the electroactive bacteria, might degrade the organic matter or release e^−^ into the surrounding vicinity; if the released e^−^ was in the distance to reach anode or conductive biofilm, e^−^ might be collected. FAB might contribute to these processes due to increased anode biofilm thickness. For example, You et al. (2015) noted that suspended microbes, even if not generated e^−^, might degrade the organic matter that EAB can later consume. In contrast, as the thickness increases further, the e^−^ transfer might be affected. Current studies indicate metal-like conductivity in the EABs and e^−^ conductivity about a centimeter distance from the electrode (Malvankar et al. 2011; Yuan et al. 2020). However, this idea remains debatable as a practical means to prove the hypothesis in a ongoing research (Strycharz and Tender 2012).

Against the hypothesis, in the anaerobic-MFC system (MFC-3), the immense benefit of FAB thick anode biofilm seems ineffective to improve e^−^ collected at the anode. Unlike MFC-1 and -2, in -3 the power output and CE becomes negligible (*p* > 0.05) between FAB and MFC. This inconsistency might occur due to insufficient fuel to supply the developed thick anode biofilm (Fuel/EAB) (i.e., it may create an inactive zone) and corresponds with the anode to volume ratio (*A*/*V*). These results indicate that the enhanced EAB growth should be examined under several pretreatments with controlled complex system stressing conditions. Otherwise, it can mislead the conclusion that enhanced EAB formation could not boost power yield. Probably, this might be a remarkable reason that obscured a milestone power output and CE achievement in previous studies.

Voltage drops at the end of each treatment cycle and the onset of the consequent phase. Similar patterns were reported elsewhere (Su et al. 2013; Ye et al. 2019). Voltage perturbation was more recurrent in the aerobic-MFC relative to the anaerobic-MFC. Hence, this result suggests the FAB system contributed more to aerobic-MFC integrated treatment than the anaerobic-MFC system. It could be due to DO shock from the aerobic reactor that destabilizes the MFC system compared with anaerobic-MFC. The causes of voltage drop in the MFC-integrated wastewater treatment system are diverse: oxygen diffusion to the anode, pH (59 mV loss/unit change), COD shock arising from the subsequent reactor to the MFC, starvation, and abiotic factors such as internal resistance (Gajaraj and Hu 2014; Oh and Logan 2007). EABs have reversible hydrogenase enzymes that switch extra cellular e^−^ release processes to capture e^−^ inside the cell, resulting in a voltage drop (He et al. 2017). It is essential to build thick anode biofilm and optimize the operational condition (He et al. 2017; Sun et al. 2016). Therefore, the thick biofilms on the FAB electrode might reduce the oxygen effect on the exoelectrogen bacteria.

Figures 7 and 8 show that the voltage generation decreases when the COD_IN_ declines except for MFC-2 in the anaerobic-MFC system. The substrate shock (fuel: COD) in both the aerobic-MFC and anaerobic-MFC was accompanied by voltage drop, but consequently, there was a variation in the voltage output. After the shock, voltage increase in the anaerobic-MFC system (MFC-2) but declines in aerobic-MFC. It might be due to oxygen intrusion from the aerobic reactor effluent, which signals within a certain COD range; the DO level could be more critical to determine the voltage output and drop. Aerobic treatment is the most efficient system for extracting the energy stored in the substrate than methanogenesis and fermentation; Gibbs free energy change (∆*G*^0^) for glucose oxidation was − 2882, − 428, −342 kJ/mol, respectively (Comeau 2008). Hence, aerobic pretreatment, low COD_IN_, high DO, and inoculant nature might cause the MFC-2 decrease in aerobic-MFC than the anaerobic-MFC system.

The maximum coulombic efficiency (CE) observed in anaerobic-MFC (0.91%) was higher than in the aerobic-MFC system (0.80%) (Table 2). Similar ~ 1% CE was recorded by Zhang et al. (2009), but a higher CE (3–12%) was noted by Liu and Logan (2004). Lower CE is an issue in real wastewater treatment using MFC (Lu et al. 2009). Cofactors may result in CE loss: aerobic pretreatment (lower COD_IN_) and several final e^−^ acceptors (DO, ${\text{NO}}_{3}^{ - }$, and ${\text{SO}}_{4}^{2 - }$). Anaerobic processes such as methanogenic and anaerobic ammonia oxidation (Anammox) may shift the e^−^ pathway and reduce CE. The DO may result in additional harm to the strict anaerobic EAB. The CE difference between FAB and MFC was negligible (0.01–0.12%); this might be ∆COD could not be differentiated because both systems were operated in the same reactor. In another optimization setup (data not shown), FAB and MFC were placed in a separate reactor, and FAB improved CE by ~ 10% beyond MFC. However, this could be a potential research question for future studies.

At a steady state, the COD_EF_ in the solitary MFC was above 200 mg/L. In contrast, MFC-integrated system reduces COD_EF_ below 125 mg/L, which was 82–124 mg/L (MFC-2) and 52–27 mg/L (MFC-3) (Fig. 5). Standalone MFC requires further treatment to meet the COD_EF_ discharge standard, 50 mg/L (China) and 20 mg/L (Korea) (Yu et al. 2012). This finding was in agreement with Ren et al. (2014), MFC treated domestic WWT COD_EF_ ranges 23–164 mg/L (fed-batch) and 60–220 mg/L (continuous) based on HRT, reactor configuration, and COD_IN_. When the pretreatment increases (1–2 days) at the same HRT of 1 day in MFC, the COD_EF_ declines. It might be due to the diverse microbial population contributing to the organic matter degradation, extended HRT, or enhanced nutrient mixing during substrate was transferred from one reactor to another. The COD_IN_ fluctuation on day 12 (MFC-2) was caused by sludge resuspension, stabilized after the flow rate was restored. The experimental data support the novel FAB–MFC system better reduce voltage drop than MFC; however, the COD_RE_ could not be concluded because both FAB and MFC systems were inserted in the same reactor and operated under similar near anodic pH. Nevertheless, the aerobic-MFC COD_RE_ outweighs the anaerobic-MFC system. Overall, the MFC-integrated system COD_RE_ was (78–97%) higher than the solitary MFC (68–78%) treatment. These COD_RE_ results are in line with other studies on MFC-integrated systems (66–97%) (Chen et al. 2019; Gajaraj and Hu 2014) and standalone MFC (22–80%) (Kim et al. 2016; Liu and Logan 2004) (Table 2). In MFC, COD_RE_ depends on COD_IN_ type; from the total COD (tCOD) biofilms prefer the soluble COD (sCOD) than particulate COD (pCOD) (Ren et al. 2014). Hence, this study elevated COD_EF_ could be due to higher pCOD in the system.

The COD_RE_ and CE during the treatment period are shown in Fig. 6. As shown in the figure, in MFC, the COD_RE_ increases with an integrated system, MFC-1 < -2 < -3. In particular, the COD_RE_ in aerobic and anaerobic-MFC integrated system was 71–74% in MFC-1 (COD_IN_ = 755 mg/L), 75–78% in MFC-2 (COD_IN_ = 456–500 mg/L), and 84–85% in MFC-3 (COD_IN_ = 265–351 mg/L). CE decreases with COD removal increases. CE bears a resemblance to electricity generation than COD_RE_. The cause behind inverse CE and COD_RE_ association is unknown (Yu et al. 2012); however, the factors might be e^−^ loss in the system due to endoelectrogens or biofilm conductivity problems. Carbon (e^−^) balance study implicates ${\text{SO}}_{4}^{2 - }$ reduction (37–64%) is the major e^−^ scavenger followed by methanogens (1.3–3%) (Zhang et al. 2013).

Additional MFC-integrated system advantage could be stabilized influent to the electroactive bacteria (SD = 12–23 mg/L COD_IN_), relative to (raw WW) direct-fed MFC (SD = 52 mg/L COD_IN_). This stability may arise from influent steady-state nature: partially treated, anaerobic, and intermediate metabolites. In contrast, You et al. (2015) reported stability of standalone MFC (anodic biofilm) under different feedstock conditions. This variation could arise from DO during pretreatment in this study, while the authors change the substrate (acetate and casein).

The improved anode surface area (ASA) should support the EAB life cycle, an overlooked part in ASA modification, especially the detachment means from the electrode. The biofilm formation comprises three basic stages: attachment, maturation, and detachment from the electrode surface (Read et al. 2010). The lipopolysaccharides (LPS) and exopolysaccharides (EPS) are crucial for biofilm formation. In EAB, the EPS attaches cell to cell over the electrode—recent pieces of evidence support the electrical conductivity nature of EPS in MFC (Angelaalincy et al. 2018). According to Yu et al. (2017), one of the critical concerns of 3D electrodes fabricated using nanotechnology was the small pore size forbids interior biofilm growth. The microbial electrode pore size should not be < 100 μm as the biofilm thickness is 30–50 μm on average. This nanopore size might not develop a centimeter-long biofilm, where recent studies recommend thick anode biofilm (Malvankar et al. 2011). In addition, the electrode reusability was neglected (regenerate MFC), while the focus was on ASA and pore size improvement. Dead bacteria may attach to the electrode and interfere with MFC performance (Sun et al. 2016). Hence, as the pore size narrows, dead biomass may deposit and block material and e^─^ flow. Consequently, it becomes difficult to re-use the electrode via a simple technique such as rinsing with water, scrapping, adjusting the flow rate, or increasing the electrode cleanup cycle.

Despite further studies, the MEJ-dish approach enables thick anodic biofilm growth and easy removal of the MEJ-dish and biofilm; again, re-sizing the dish could monitor the EAB growth. However, the limitation of this study was examining the MEJ+ and MEJ− electrodes in the same reactor. For instance, ∆COD could not be differentiated between FAB+ and MFC systems. Ultimately, this effect may obstruct the performance difference between the two systems; hence, future studies might consider a separate reactor with different MEJ-dish, wastewater, or inoculum.

In general, these findings implicate, anode modification with MEJ-dish (FAB) improves the power output. Similarly, Zhou et al. (2012) noted that enhancing anode area improves the MFC performance. Against this conclusion, Di Lorenzo et al. (2010) observed anode surface increment with granulated packed graphite pellets did not increase the current output due to mass distribution. Hence, increasing ASA alone could not always ensure performance increment. Probably, the increased ASA was not suitable for the EAB growth, results in discontinuous EAB formation, fuel (substrate) could not reach the modified area as it is far away from the external surface, or narrow pore size (due to packing or nano-modification) that can be easily clogged by solids in the liquid.

### Comparison of FAB–MFC integrated systems with other studies

The MFC-integrated systems performance was compared with other studies (Table 2). The results observed in this study were comparable with previously reported findings (Chen et al. 2019). The mean COD_RE_ observed in this study (71–95%) was higher than Goto and Yoshida (2019) (49%) but closer to Liu et al. (2019) (80%) findings. However, it might be challenging to make a solid conclusion due to variation in MFC setups and COD_IN_. In addition, the previous study reactor volume ranges from 0.18 to 40 L, external load varies from 3 to 1000 Ω and MFC configuration (Goto and Yoshida 2019; Liu et al. 2019; Wen et al. 2009).

The power density observed in standalone MFC was ~ 3 times lower than in Liu and Logan (2004) because the authors used PEM (Nafion) with lower resistance than the salt bridge used in this study. For example, Min et al. (2005) noted internal resistance of PEM (1.3 kΩ) was ~ 15 times lower than the salt bridge (20 kΩ). In addition, the authors used glucose substrate, but real wastewater has several e^−^ acceptors that lower the power efficiency. On the other hand, the power density observed in this study was higher than 0.3 mW/m^2^ by Min et al. (2005) using a salt bridge and pure culture (*G. metallireducens*). Power output variation may arise from the inoculum (Ishii et al. 2017; Santoro et al. 2017; Xu et al. 2016). Even the mechanism of salt bridge synthesis affects the power output; as Sevda and Sreekrishnan (2012) noted, increasing salt concentration up to 5% raises proton transfer capacity, lowers internal resistance, and improves the power output by ~ 11 times. The power output observed in this study was ~ 4 times higher than that in Rodrigo et al. (2007); since information on salt bridge synthesis was not provided, further discussion could not be made.

Compared to the standalone MFC (MFC-1), the aerobic-MFC integrated (MFC-2 and -3) system generated lower power output. The cause might be the aerobic treatment that reduces the MFC influent fuel concentration or results in DO contamination. But, the anaerobic-MFC system influent prompts MFC-2 beyond MFC-1 and -3. Overall, anaerobic influent better stabilizes influent and enhances the performance. Similar effects were reported by Chen et al. (2019); Li et al. (2015); and Yadav et al. (2012). In contrast to Bose et al. (2018); Kalathil et al. (2013﻿); and Rodrigo et al. (2007), the present study did not apply aeration to the cathode. Another advantage of this system is using a hybrid cathode, oxygen in the air, and tap water as an e^−^ acceptor. If the hybrid-cathode were not used, aeration might be required. Vicari et al. (2016) observed 81% of DO (3–0.57 mg/L) was consumed within less than an hour in the cathode chamber. That is why the reported D-MFC with oxygen as e^−^ acceptor was obliged to aerate the cathode chamber (Table 2).

### Applications of FAB–MFC

This study presented a simple, practical technique (FAB) to increase anode surface area that influences the anode biofilm structure without chemical or thermal modification. It was evaluated in different MFC configuration systems. For example, metal electrodes are more e^–^ conductors, but the major limitation is microbial attachment surface area; hence, etched with sulfuric acid (Nosek et al. 2020). In the FAB reactor, the junction point between MEJ-dish and electrode is crucial. This area has a determinant limitation for additional e^−^ collection, affecting bioelectricity production, particularly at startup. In our previous study, several MEJ-dishes with different junction types: open, partly open, and closed for EAB growth were examined (Atnafu and Leta 2021﻿). The MEJ-dish (e.g., K3) with open junction space for EAB growth yields a fascinating result; naked eye-observable thick biofilm supersedes MFC voltage output (max open circuit 0.9 V) and is vital at later age of the reactor during stress such as organic matter depletion.

In contrast, the MEJ-dish covered junction point lowers voltage output at startup and extends the period to reach a steady state. One of the peculiar features we notice is that MEJ-dish might create a small pocket of a strict anaerobic zone where the EAB favors colonizing. Of course, it might attract competitors and results against the expectation. Most review papers on MFC suggest the need for innovative paradigm shift and study on electrode fabrication, configuration, and operation (Yu et al. 2017). Future studies might need to develop creative MEJ-dish to support EAB growth.

On the other hand, questions may be raised on FAB practicality. In pure culture study, the biofilm thickness was electrochemically active in ~ 20–50 μm thick (Sun et al. 2016), so why is the need to increase beyond? Even if the anode biofilm thickness increases, the electrical efficiency might be compromised due to electrode overpotential, ohmic loss, activation loss, parasitic loss, current, and mass distribution (Choudhury et al. 2017; Di Lorenzo et al. 2010)?

Addressing these issues, recent studies by Yuan et al. (2020) and Malvankar et al. (2011) indicate anode biofilm tendency to transmit e^─^ over a centimeter long. Mixed culture increases biofilm thickness and minimizes oxygen diffusion to the inner EAB layer (Yang et al. 2019) and results in a synergetic effect than pure culture (Goto and Yoshida 2019; Logan 2008). Additionally, it could be possible to manage the biofilm thickness by re-sizing the MEJ-dish. Extra electrode biofilm growth and removable cleaning part (MEJ-dish) might ease the electrode cleaning using simple techniques such as shear force and flow rate. The MFC performance strongly depends on anode geometry (Merkey and Chopp 2012). Hence, the HD-MEJ+ approach might enable a simultaneous advantage of multi-dimension electrodes in a single design.

According to Yu et al. (2021) and Zhang et al. (2013), even if MFC bioelectricity generation is not enhanced, improving treatment performance is outstanding achievement. In fact, increasing anode surface and biofilm thickness alone, to any required degree, could not lift the electrical output unless associated factors address from power generation to collection. However, it may contribute a small step toward filling the anode biofilm growth drawbacks. Supporting the argument, Choudhury et al. (2017) suggested that novel electrode design or surface modification (physical or chemical) enhances e^−^ conductivity and bacterial adhesion. Overall, given further studies, the FAB concept introduces novel HD microbial electrode design such as T-shape electrode (MEJ-dish + electrode = flexible hybrid 3D electrode) rather than plain, flat, rod-shaped, or fixed 3D electrode (carbon foam/brush) that could dominate the current MFC research.

## Conclusions

In this study, a novel fragmented anode biofilm microbial fuel cell (FAB–MFC) integrated system was developed and investigated for domestic wastewater treatment and bioelectricity generation. The anode with microbial electrode jacket dish (MEJ-dish) was designed to enhance anode biofilm growth and system performance. FAB (MEJ+) and MFC (MEJ−) were compared for power output performance. The FAB enabled variable (thick and thin) biofilm formation compared to MFC. The FAB simple, straightforward technique increases anode biofilm thickness ~ 5 times a bare electrode. The FAB–MFC (FAB+) integrated system improved the COD removal compared with solitary MFC. However, it was impossible to conclude the FAB+ ∆COD and CE efficiency because both electrodes (MEJ+ and MEJ−) were inserted in the same reactor. The MFC-integrated system power generation was affected with the pretreatment level for < 800 mg/L COD_IN_ at 0.76 kg-COD/m^3^/day load. The anaerobic-MFC integrated system power generation was found significantly higher than the aerobic-MFC. The bioelectric generation was greater in solitary (directly fed) MFC than in aerobically treated effluent-fed MFC. The FAB system generates the highest power than MFC in anaerobic-MFC (FAB = 104 mW/m^2^, MFC = 98 mW/m^2^) and aerobic-MFC (FAB = 59 mW/m^2^, MFC = 42 mW/m^2^) integrated system. Voltage drops were noticed during treatment phase transition, and FAB reduces the voltage drop relative to MFC. The FAB+ integrated system could be applied for real applications and enhance performance. It might depend on the substrate (COD) load, DO concentration, and microbial diversity in the inoculum. Hence, further studies will be required to understand the FAB+ efficiency in terms of inoculant nature, MEJ-dish type, and electrical conductivity over thick long-distance biofilm.

### Supplementary Information

**Additional file 1: Fig. S1.** The proposed hypothetical presentation of the FAB conceptual model to form thick anode biofilm. **Fig. S2.** Screening (1), sedimentation (2), and anaerobic reactor (3). **Fig. S3.** The detailed schematic diagram of the aerobic reactor (R4). **Fig. S4.** Schematic diagram of H-type air diffuser (a) designed and (b&c) constructed. Drawings are not to scale. The diffuser was inserted into the aerobic reactor (MBBR). **Fig. S5.** The FAB-MFC integrated system (a-c) during construction and (d) photo. **Fig. S6.** The methanogenic reactor (a) schematic diagram and (b) photo. **Fig. S7.** Ball valve (a) PPR, (b) Brass, (c) PVC, and (d) reactors stand support. **Fig. S8.** Microbial electrode jacket-dish (designed). D = dimension. **Fig. S9.** Schematic diagram of the microbial fuel cell (MFC) integrated domestic wastewater treatment system options (1-3). **Fig. S10. **Observed biofilm on the MEJ+ electrode.

## Data Availability

The data sets used in this study are available from the corresponding author on reasonable request.

## Abbreviations

ASA: Anode surface area
MFC: Microbial fuel cell
MEJ: Microbial electrode jacket
FAB: Fragmented anode biofilm
