# Supplementary material for: A novel fragmented anode biofilm microbial fuel cell (FAB–MFC) integrated system for domestic wastewater treatment and bioelectricity generation
Source: Bioresour Bioprocess. 2021 Nov 13;8(1):112. doi: 10.1186/s40643-021-00442-x (PMC10991661; doi:10.1186/s40643-021-00442-x)
Supplement: Supplementary file 1 — Additional file 1: Fig. S1. The proposed hypothetical presentation of the FAB conceptual model to form thick anode biofilm. Fig. S2. Screening (1), sedimentation (2), and anaerobic reactor (3). Fig. S3. The detailed schematic diagram of the aerobic reactor (R4). Fig. S4. Schematic diagram of H-type air diffuser (a) designed and (b&c) constructed. Drawings are not to scale. The diffuser was inserted into the aerobic reactor (MBBR). Fig. S5. The FAB-MFC integrated system (a-c) during construction and (d) photo. Fig. S6. The methanogenic reactor (a) schematic diagram and (b) photo. Fig. S7. Ball valve (a) PPR, (b) Brass, (c) PVC, and (d) reactors stand support. Fig. S8. Microbial electrode jacket-dish (designed). D = dimension. Fig. S9. Schematic diagram of the microbial fuel cell (MFC) integrated domestic wastewater treatment system options (1-3). Fig. S10. Observed biofilm on the MEJ+ electrode. [file 40643_2021_442_MOESM1_ESM.docx]

# **Supplementary information**

**A novel fragmented anode biofilm microbial fuel cell (FAB-MFC) integrated system for domestic wastewater treatment and bioelectricity generation**

Tesfalem Atnafu^a,b,^^[[1]](#footnote-1)^*, and Seyoum Leta^a^

^a^ *Center for Environmental Science, Addis Ababa University, Addis Ababa, Ethiopia*

^b^ *Department of Biological Science, College of Natural Sciences, Mettu University, Mettu, Ethiopia*


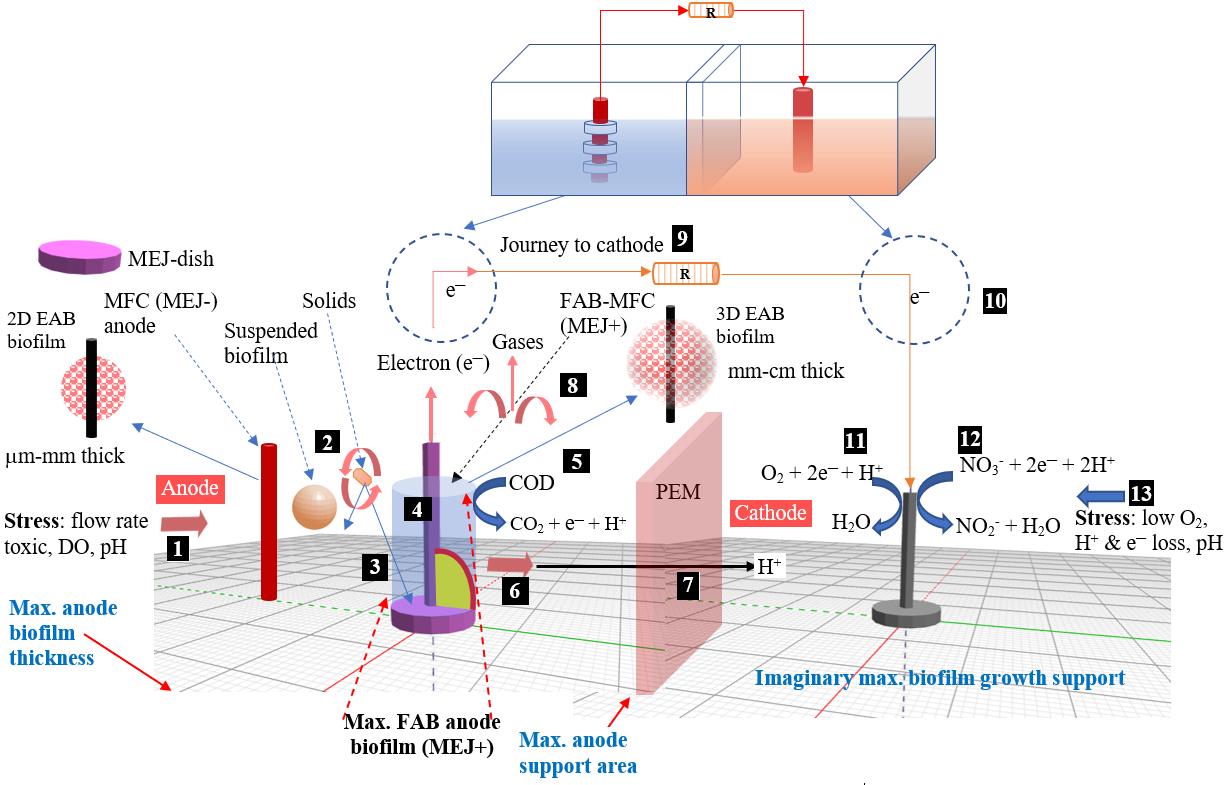


**Fig. S1.** The proposed hypothetical presentation of the FAB conceptual model to form thick anode biofilm. Where R resistance, PEM permeable membrane, Max. maximum, FAB fragmented anode biofilm, MEJ-dish microbial electrode jacket dish, EAB electroactive biofilm, 2D and 3D two and three-dimension, in turn.

**FAB schematic conceptual model**

= Flow rate, DO, pH variation, and wastewater composition could affect MFC. For instance, the wastewater content might affect the microbial activity, e.g., toxic chemicals, heavy metals, and antibiotics (Liu and Cheng 2014).

**1**

= Suspended biofilm or solids effect (Santoro et al. 2017).

**2**

= FAB thick anode biofilm. Microbial electrode dish (MEJ-dish) supporting the anode biofilm growth. The electrode materials affect bacterial adhesion, e^─^ transfer, and electrochemical efficiency (Abbassi et al. 2020). MFC architecture paradigm shift is required to ensure maximum (max.) contact between the substrate and the biofilm, enhance biofilm thickness, and a higher electrode surface to electrolyte volume ratio (Abbassi et al. 2020; Chaturvedi and Verma 2016; Choudhury et al. 2017; Do et al. 2018; Flimban et al. 2019).

**3**

= Electron transfer to anode: Interaction or coexisting factors during e^─^ transfer between bacteria and solid electrodes is not well described, especially in complex environments where many microbial species (electroactive or not) can be found on the electrodes (He et al. 2017).

**4**

= Microbial oxidation of organic matter (Logan et al. 2006).

**5**

= Internal resistance (R_int_). As the size of the MFC increases, the system R_int_ increases, which results in lower power density (PD). According to Logan and Regan (2006), decreases in a system R_int_ increase the system PD, which enhances electricity generation (Abbassi et al. 2020).

**6**

= Internal resistance (R_int_) of the proton exchange membrane (PEM). H^+^ diffusion to the cathode via PEM: oxygen reduction reaction (ORR) remains the main MFC technology bottleneck due to high over-potentials and low kinetics encountered (Santoro et al. 2017). PEM should avoid minerals, oxygen, and substances transfer between the chambers (Rahimnejad et al., 2014), lower R_int_, and cover ~ 40% of the total cost (Abbassi et al. 2020). For example, Min et al. (2005) observed the R_int_ of a salt bridge (20 kΩ) was higher than Nafion (1.3 kΩ), and Nafion increases PD by 40 mW/m^2^ than a salt bridge. Substrate crossover in the cathode compartment affects the MFC performance negatively (Koroglu et al. 2019).

**7**

= Gases or other biochemical byproducts (Pant et al. 2012).

**8**

= External load or resistance (R_ext_) (Choudhury et al. 2017; Logan et al. 2006).

**9**

= The distance between anode and cathode (Logan et al. 2006). Decreasing the distance between anode and cathode decreases the R_int,_ especially in membrane-less MFC (Ahn and Logan 2013). For example, R_int_ decrease from 35 (at 2 cm) to 16 Ω (at 1 cm) when distance decrease. However, when the distance decrease further could results in a short circuit or oxygen diffusion from cathode to anode. The anode and cathode distance could have a vice versa or reverse effect on MFC efficiency. Hence, it requires a coordinated solution to address the challenges.

**10**

= Chemical reduction (Koroglu et al. 2019; Santoro et al. 2017). The cathode region is the major MFC shortcoming. A lower concentration of terminal e^─^ acceptor at the cathode limits e^─^ flow. Most MFCs depend on the dissolved oxygen (DO) present at the cathode as e^─^ acceptors (Zhao et al., 2006). Maintaining high bulk concentrations and distributing oxidants such as O_2_ across the cathode compartment can reduce mass transfer losses (Koroglu et al. 2019). It occurs when the species’ mass transport rate to or from the electrode limits current production.

**11**

= Microbial reduction (Santoro et al. 2017).

**12**

= Stress affecting the cathode chamber (He et al. 2017; Santoro et al. 2017).

**13**

**Experimental setup design and construction**

1. **Screening tank (R1)**


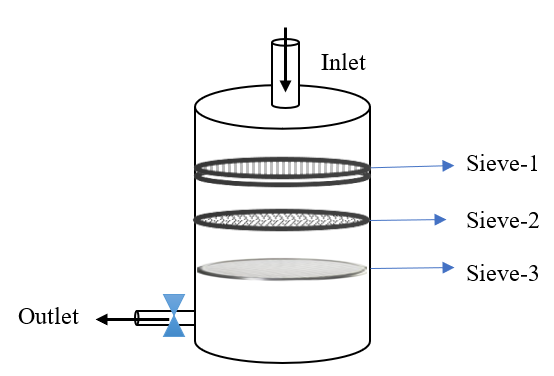

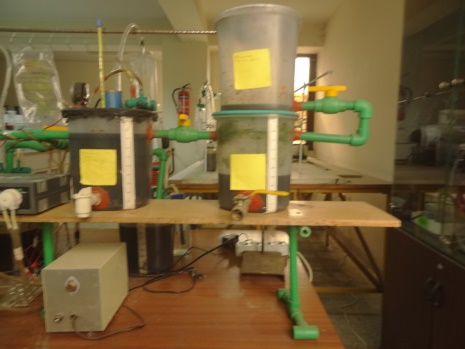


R3

R2

R1

1. **Sedimentation tank (R2)**


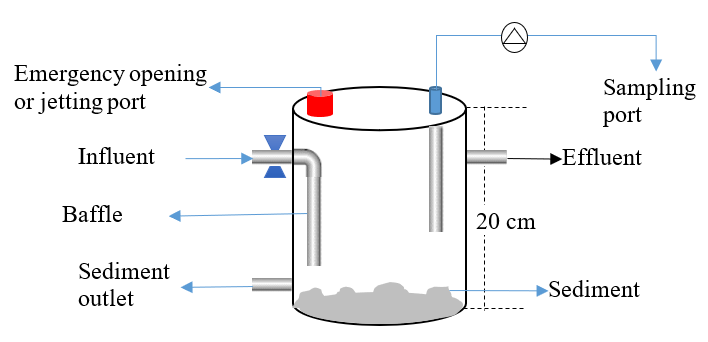

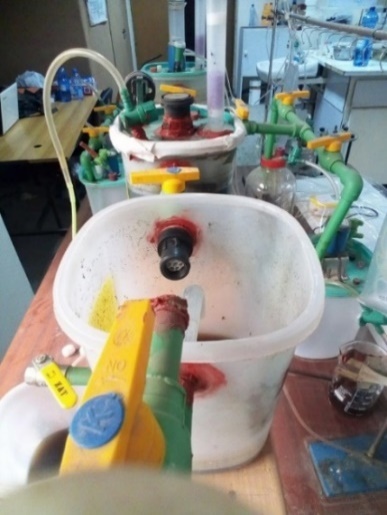

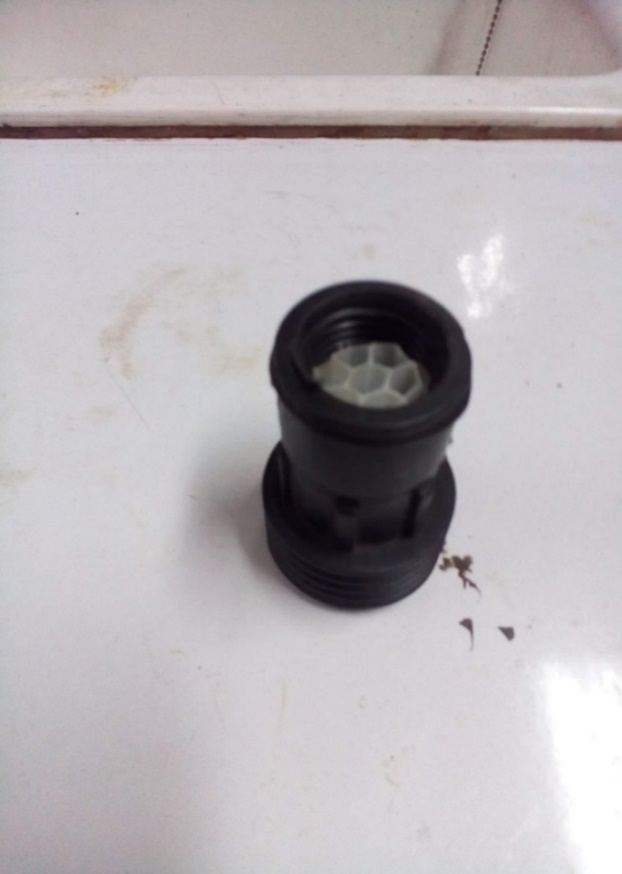


1. **Anaerobic reactor (R3)**


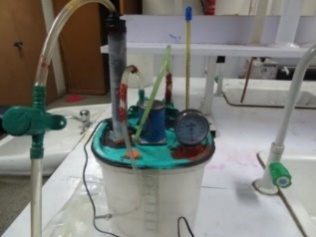
Pressure gauge (Bio-pluss™) and thermometer (Abron) that measure from -50 to 150 ℃ were top-mounted on the anaerobic reactor.


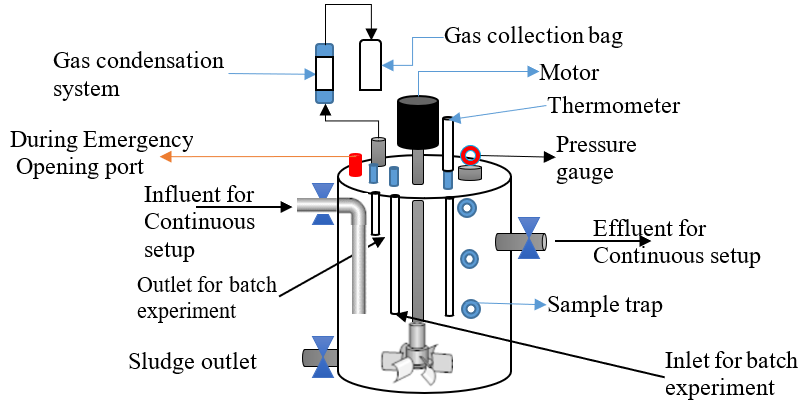

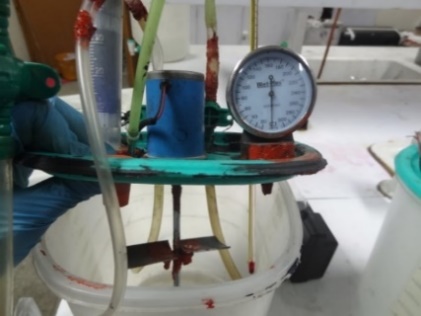


**Fig. S2.** Screening (1), sedimentation (2), and anaerobic reactor (3).

1. **Aerobic reactor design (R4)**

The aerobic reactor was designed based on the conventional activated sludge process. The aerobic reactor was equipped with (4.1) Aerobic tank, (4.2) Airflow system with blower instruments, (4.3) Biofilm growth support structure, (4.4) Sampling traps, and (4.5) Sensor’s inlet for measuring the physicochemical parameters (pH, DO, and temperature).


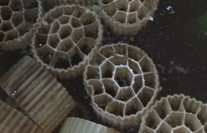


- 1. **Aerobic tank**


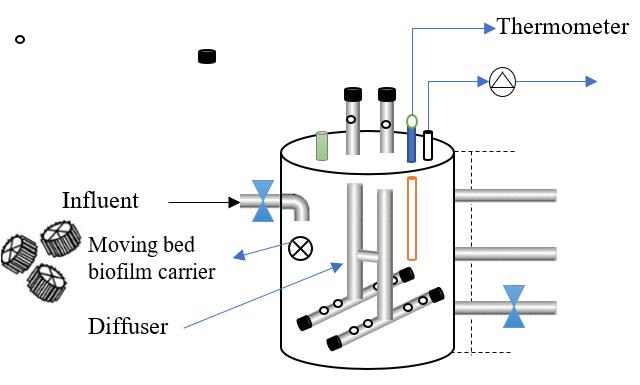

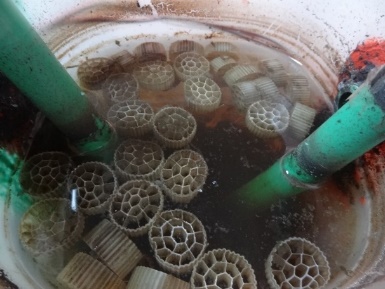


**Fig. S3.** The detailed schematic diagram of the aerobic reactor (R4).

- 1. **The aeration system**

In this research, from the different aeration systems (point injection diffuser, multiple injection diffuser, jet aerator, and submerged turbine aerator JSWA (2013)), point injection diffuser was selected and modified the diffuser distributes the inlet air uniformly across the reactor. The aeration provides uniform airflow throughout the reactor from all the release points.


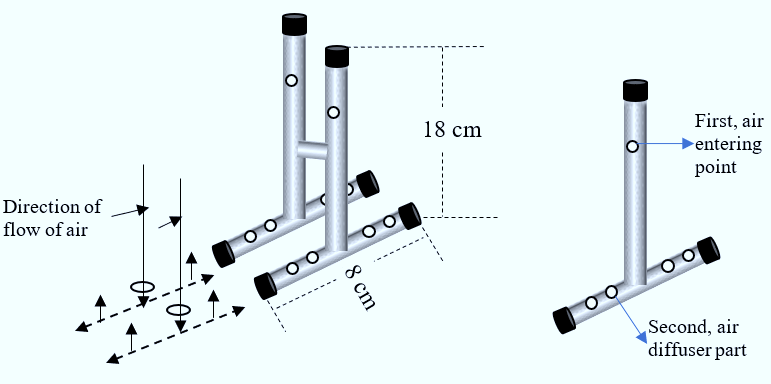


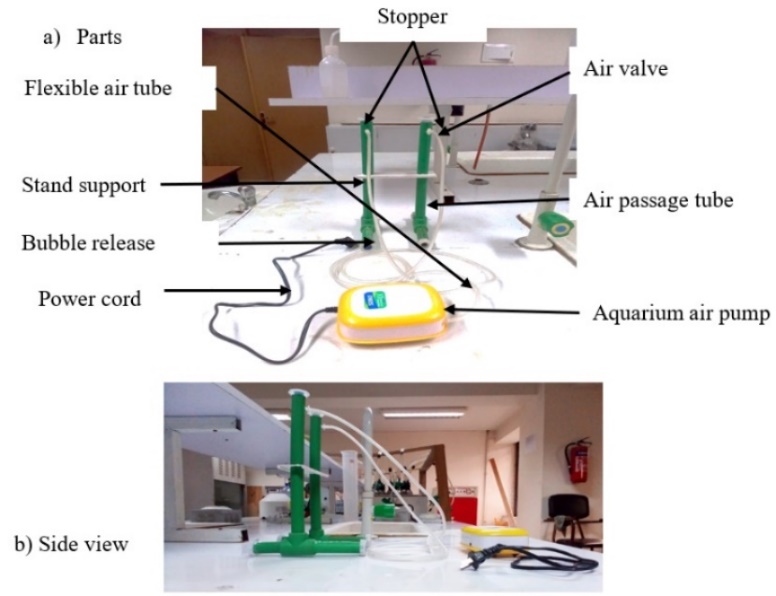


a)

Air flow direction

b) Side view


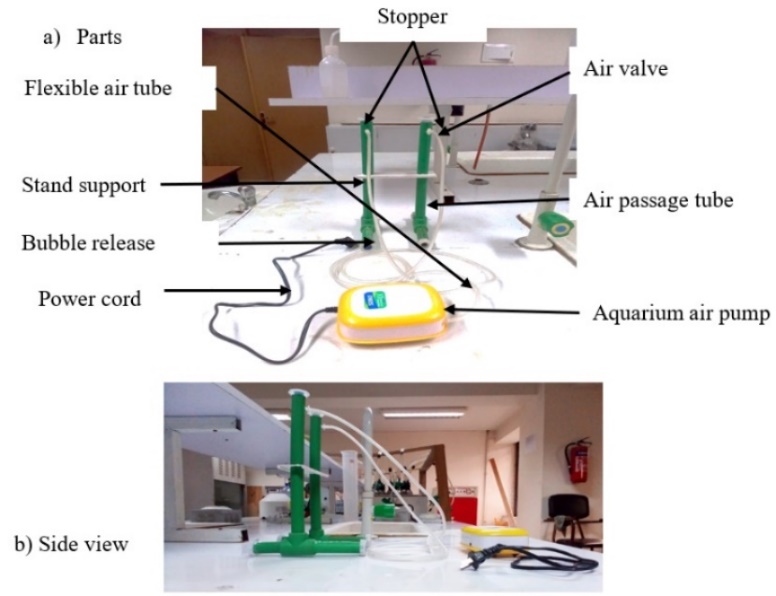


c)

**Fig. S4.** Schematic diagram of H-type air diffuser (a) designed and (b&c) constructed. Drawings are not to scale. The diffuser was inserted into the aerobic reactor (moving bed biofilm reactor=MBBR).

1. **Microbial fuel cell reactor (R5/7)**


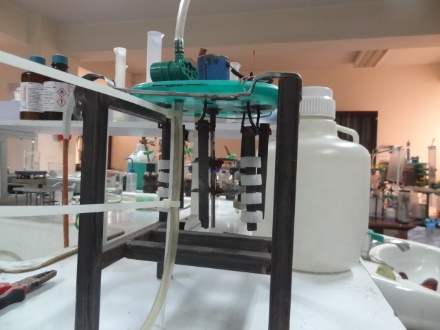

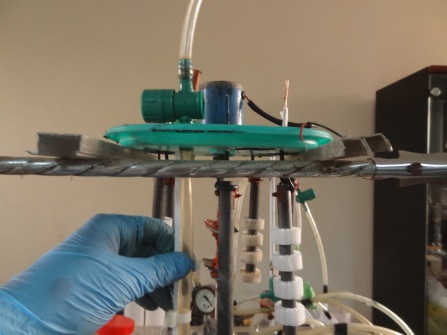


b)

a)


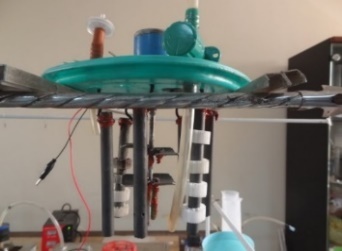

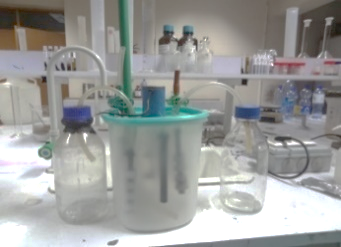


d)

c)

FAB-MFC

Motor

Cathode

**Fig. S5.** The FAB-MFC integrated system (a-c) during construction and (d) photo.

1. **Methanogenic reactor (R6/8)**


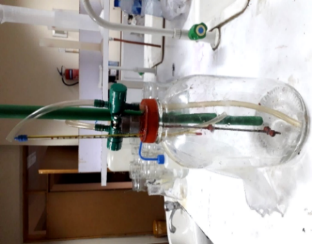


b)

Biogas

Sample inlet

N_2_ sparging

Impeller

Shaft

Driver motor

Methanogenic

Reactor


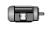

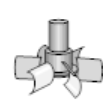


a)

**Fig. S6.** The methanogenic reactor (a) schematic diagram and (b) photo.


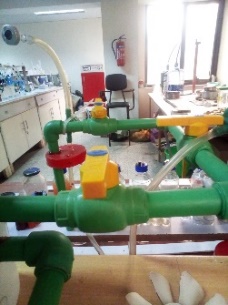

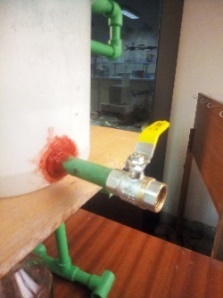

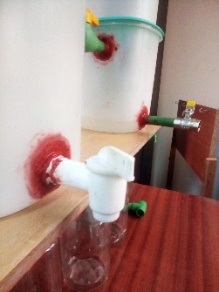

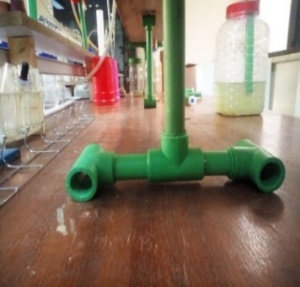


a)

c)

b)

d)

**Fig. S7.** Ball valve (a) PPR, (b) Brass, (c) PVC, and (d) reactors stand support.

MEJ+

FAB-MFC or FAB+

MEJ-

MFC

MEJ-dish

2D

3D

3D

3D

2D

2D

Hybrid dimension (HD)

**Fig. S8.** Microbial electrode jacket-dish (designed). D = dimension.

**Experimental setup operation**


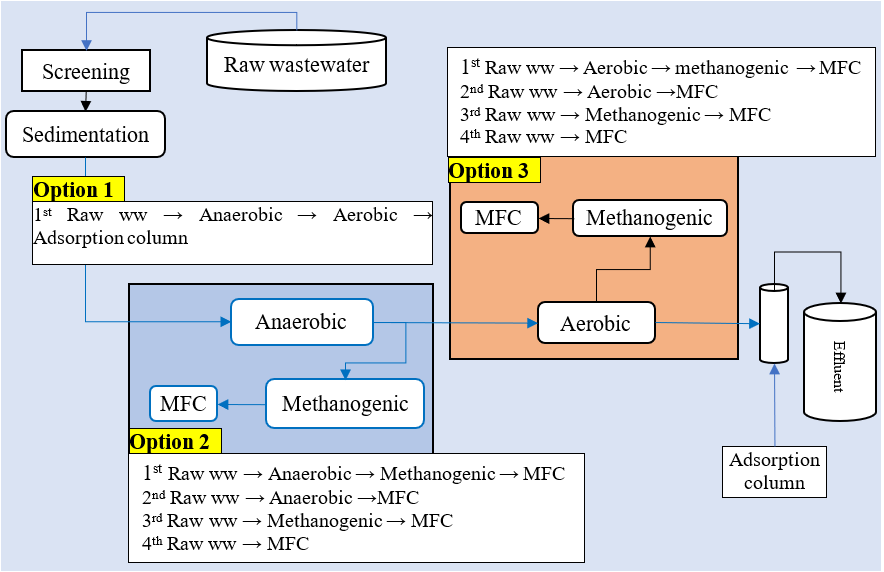


**Fig. S9.** Schematic diagram of the microbial fuel cell (MFC) integrated domestic wastewater treatment system options (1-3).

As shown in Fig. S8, the integrated system consists of three major operation parts: the treatment (option 1) and the MFC integrated system (option 2 and 3). Additionally, options 4, 5, and 6 were identified (but not shown in Fig.S8). In this study, only options 2 and 3 were investigated. The liquid contents were mixed using an overhead mounted DC motor (24 V) to keep even biomass distribution in the reactors. Each system was operated for more than 30 days before evaluating the performance. The reactors were inoculated with mixed culture and worked in fed-batch mode at room temperature (25 ± 1 ℃) without sludge returning, pH adjustment, or nutrient addition. All the systems were considered steady-state when voltage output was less than 0.01 V/h variation under 1000 Ω external load. In all the reactors, maintenance was conducted daily. After feeding the reactors or sampling, all the reactors were checked, and if non-functioning valves or pipes were detected immediately fixed. Again, during operation, all the reactors were checked routinely and maintained as necessary. The cleanup, checkup, and maintenance were conducted manually.

All experimental cells had a sensor plugging port, except the screening (R1) and sedimentation (R2) chamber. Pressure gauge (Bio-pluss™) was top mounted on the anaerobic reactor, while thermometer (Abron Co., China) (-50 to 150 ℃) and a 24 V brushless DC motor with pinched impeller (agitator) was installed on all reactors except R1 and -2. A power flow was adjusted using a power supply unit (BK-1502DD, BAKU, China). Gasbags were attached over the anaerobic and MFC reactor. All ports were closed with a butyl rubber stopper unless otherwise pipes or valves were inserted. All openings (lid, valve, and pipe junction points) were closed and sealed using a gasket maker (ABRO Inc., USA).


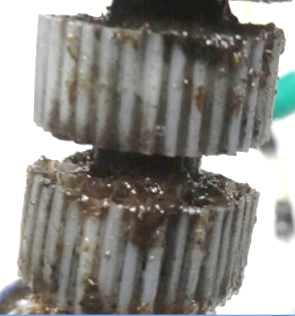

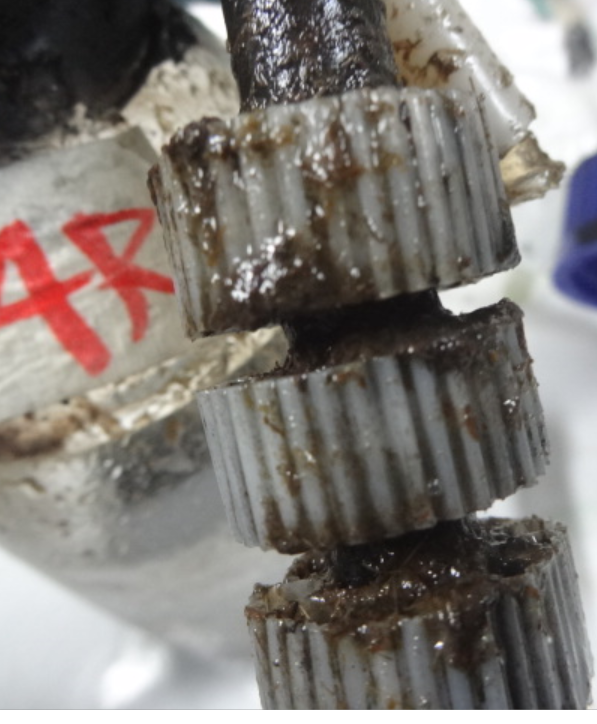


c

b

a

The (a) extended biofilm grown on the MEJ-dish, (b) biofilm formed adjacent to MEJ-dish, (c) anode electrode or graphite rod. Samples were collected from the MEJ+ and electrode to confirm the biofilm growth with a microscope and microbial dynamics using 16S rRNA and biochemical test. The extended biofilm may not directly contribute electrons to the FAB-MFC due to long-distance (2.5 cm+). Still, it may assist in degrading organic matter and provide intermediate metabolites to the biofilms within reach of the anode. They could degrade and release e^─^ and H^+^ to the anode biofilms, shift e^─^ scavenger’s competition site, H^+^ may be transported to the cathode and primarily face harsh conditions to reduce the effect on the anode biofilms. Torres et al. (2008) pointed the challenge of fuel and waste matter transport in thick biofilms. The MEJ-dish concept may assist in addressing the challenge by making micro/macropores in the MEJ-dish as a channel for mass transport. The observed data implicate, it could be possible to propose that the MEJ-dish is working as an electroactive biofilm culture dish (plate). By adjusting the MEJ-dish, it may be possible to lower or enhance the thickness. However, this needs further study.

**Fig. S10.** Observed biofilm on the MEJ+ electrode.

**Supplementary information references**

Abbassi R, Yadav AK, Khan F, Garaniya V (2020) Integrated microbial fuel cells for wastewater treatment. Butterworth-Heinemann, MA, United States. <https://doi.org/10.1016/C2017-0-03157-9>

Ahn Y, Logan BE (2013) Domestic wastewater treatment using multi-electrode continuous flow MFCs with a separator electrode assembly design. Applied Microbiology and Biotechnology 97(1):409-416. <https://doi.org/10.1007/s00253-012-4455-8>

Chaturvedi V, Verma P (2016) Microbial fuel cell: a green approach for the utilization of waste for the generation of bioelectricity. Bioresources and Bioprocessing 3(1). <https://doi.org/10.1186/s40643-016-0116-6>

Choudhury P, Uday USP, Bandyopadhyay TK, Ray RN, Bhunia B (2017) Performance improvement of microbial fuel cell (MFC) using suitable electrode and Bioengineered organisms: A review. Bioengineered 8(5):471-487. <https://doi.org/10.1080/21655979.2016.1267883>

Do MH et al. (2018) Challenges in the application of microbial fuel cells to wastewater treatment and energy production: A mini review. Sci Total Environ 639910-920. <https://doi.org/10.1016/j.scitotenv.2018.05.136>

Flimban SGA, Ismail IMI, Kim T, Oh S-E (2019) Overview of recent advancements in the microbial fuel cell from fundamentals to applications: design, major elements, and scalability. Energies 12(17):3390. <https://doi.org/10.3390/en12173390>

He L, Du P, Chen Y, Lu H, Cheng X, Chang B, Wang Z (2017) Advances in microbial fuel cells for wastewater treatment. Renewable and Sustainable Energy Reviews 71388-403. <https://doi.org/10.1016/j.rser.2016.12.069>

JSWA (2013) Design standard for municipal wastewater treatment plants, 2nd edn. Japan Sewage Works Association (JSWA), Tokyo, Japan

Koroglu EO, Yoruklu HC, Demir A, Ozkaya B (2019) Scale-up and commercialization issues of the MFCs: challenges and implications. In: Microbial electrochemical technology. Elsevier, pp 565-583

Liu W-F, Cheng S-A (2014) Microbial fuel cells for energy production from wastewaters: the way toward practical application. Journal of Zhejiang University SCIENCE A 15(11):841-861. <https://doi.org/10.1631/jzus.a1400277>

Logan BE et al. (2006) Microbial fuel cells:  methodology and technology. Environ Sci Technol 40(17):5181-5192. <https://doi.org/10.1021/es0605016>

Min B, Cheng S, Logan BE (2005) Electricity generation using membrane and salt bridge microbial fuel cells. Water Res 39(9):1675-1686. <https://doi.org/10.1016/j.watres.2005.02.002>

Pant D, Singh A, Van Bogaert G, Olsen SI, Nigam PS, Diels L, Vanbroekhoven K (2012) Bioelectrochemical systems (BES) for sustainable energy production and product recovery from organic wastes and industrial wastewaters. Rsc Advances 2(4):1248-1263.

Santoro C, Arbizzani C, Erable B, Ieropoulos I (2017) Microbial fuel cells: from fundamentals to applications. A review. J Power Sources 356225-244. <https://doi.org/10.1016/j.jpowsour.2017.03.109>

Torres CI, Kato Marcus A, Rittmann BE (2008) Proton transport inside the biofilm limits electrical current generation by anode-respiring bacteria. Biotechnology and Bioengineering 100(5):872-881. <https://doi.org/10.1002/bit.21821>

1. * Corresponding author. *E-mail address*: [tesfalem_atnafu@yahoo.com](mailto:tesfalem_atnafu@yahoo.com) (T. Atnafu). [↑](#footnote-ref-1)
